# The psychological impact of living with peritoneal mesothelioma: An Interpretative Phenomenological Analysis

**DOI:** 10.1177/13591053241298932

**Published:** 2024-11-25

**Authors:** Benjamin Lond, Lindsay Apps, Kerry Quincey, Iain Williamson

**Affiliations:** De Montfort University, UK

**Keywords:** interpretative phenomenological analysis, mesothelioma, patient experience, peritoneal, psycho-oncology, qualitative methods

## Abstract

Peritoneal mesothelioma is a rare life-limiting cancer that is likely to have an extremely negative impact on mental health; however, no studies to date have explored the impact and needs of those living with the condition. Ten individuals diagnosed with peritoneal mesothelioma (eight women, two men) participated in interviews and could share and discuss photographs to convey their illness experiences. Data analysis was informed by Interpretative Phenomenological Analysis. Two themes are presented: ‘Experiences of Care’ and ‘Psychological Distress’. Individuals experienced a lengthy diagnostic journey with little follow-up support. Women also reported negative impacts on body image due to abdominal swelling and scaring, diminished sexual ability and loss of fertility. Individuals recalled vivid feelings of anxiety and post-traumatic stress, and tried to cope by compartmentalising their fears and modifying diets. These findings demonstrate the need to further signpost services, help individuals manage gendered issues, and alleviate feelings of anxiety.

## Introduction

Mesothelioma is a rare life-limiting cancer which develops within the mesothelial cells lining either the lungs, abdomen, heart or testes. Pleural mesothelioma is the most prevalent and well-known form of the disease and typically develops within the lungs of older men as the result of prior occupational asbestos exposure. Peritoneal mesothelioma which affects the abdomen is the second most prevalent form and accounts for around 7%–10% of all mesothelioma cases (Alexander and Burk, 2016). This suggests that approximately 250–270 new peritoneal mesothelioma cases are diagnosed in the U.K. each year, though precise figures are unknown (Mesothelioma UK, 2024).

In contrast to pleural mesothelioma which mainly affects older men aged 75 and over (NHS, 2022), peritoneal mesothelioma affects women and men equally (Enomoto et al., 2019; Moolgavkar et al., 2009), and median age at diagnosis is around 50 years although the condition can develop in teenagers and the elderly (Helm et al., 2015). Peritoneal mesothelioma can spread quickly and extensively throughout a person’s abdominal cavity, and typically presents with non-specific symptoms that include: abdominal swelling and pain, fatigue, weight loss, difficulty swallowing and loss of appetite (García-Fadrique et al., 2017; Kim et al., 2017); accordingly, it is often diagnosed at a later stage when the disease burden is high and treatment options are limited (Razzak et al., 2022; Vidal et al., 2022). However, advances in cytoreductive surgery to remove visible cancer cells and chemotherapy has seen an increase in the overall survival of peritoneal mesothelioma patients (Calthorpe et al., 2023), with 5-year overall survival at 20.3% (Ullah et al., 2022).

Despite these advancements, peritoneal mesothelioma is still hard to treat and is often fatal long-term (Alexander and Burk, 2016; Greenbaum and Alexander, 2020). Consequently, the condition is likely to have an extremely negative psychological impact on affected individuals due to the acute ‘burden of disease’, a term that in this context typically refers to the days lost to illness, the disabling nature of the illness, as well as experiences of suffering and impaired quality-of-life. This is the case for people living with pleural mesothelioma who experience very high symptom burden and reduced quality-of-life (Kao et al., 2013; Nagamatsu et al., 2018; Nowak et al., 2004). Furthermore, peritoneal patients can experience temporarily lower quality-of-life after surgery and may benefit from psychological health interventions (Ali et al., 2020). To date however, psychological research has mainly focused on those with pleural mesothelioma; moreover, there is currently no literature addressing the particular psychological impacts and support needs of individuals living with peritoneal mesothelioma (Lond et al., 2022; Sherborne et al., 2024).

The current paper aims to address this research gap by providing a focused and in-depth exploration of peoples’ experiences living with peritoneal mesothelioma, in order to inform therapeutic interventions and clinical practice. This paper thus tries to answer the following research question: what are some of the experiences and support needs of individuals living with peritoneal mesothelioma, especially those differentiated from the more common pleural form of the disease?

## Methods

### Design

The design of this study was informed by Interpretative Phenomenological Analysis (IPA), a qualitative approach to research that seeks to investigate how individuals reflect on, and make sense of, their own lived experiences (Smith et al., 2022). IPA is conceptually rooted within the epistemological knowledge of human experience and is used to explore and interpret peoples’ subjective accounts to generate an in-depth understanding of the ways they experience particular phenomena (Smith et al., 2022).

Data were collected using semi-structured interviews, since they afford an ideal research space for the researcher and participant to discuss an issue at-length and co-produce a detailed experiential account on the subject (King et al., 2019). Participants were also given the option to take and share photographs as part of the data collection and interview process, to help individuals reflect on and convey their own thoughts and emotions using a visual medium which can be evocative and beneficial to the researcher becoming immersed within the participant’s own lifeworld (Williamson et al., 2024).

### Participants

Participants were recruited as part of a project exploring the experiences of people aged 60 or younger living with mesothelioma, with the current paper presenting a focused phenomenological analysis and report of the particular experiences and needs that were discussed by individuals living with peritoneal mesothelioma. In line with most types of qualitative design, IPA research typically uses a more targeted approach to recruitment that favours smaller but focused samples of experiential experts who are able to convey their in-depth experiences of an issue (Smith et al., 2022). Purposive sampling was therefore used to recruit individuals: (1) aged 18–60 years; (2) who had been diagnosed with mesothelioma; and (3) who could speak and read English – so as to engage with the research process and provide consent.

Overall, 10 individuals who had previously been diagnosed with peritoneal mesothelioma participated: eight women and two men, aged 26–59 years old, who were between 1-month and 3-years since diagnosis. All participants were White-British, heterosexual and married. Seven individuals were post-treatment (and therefore ‘cancer free’ at the time of study), two individuals were still in-treatment and one was pre-treatment and waiting to undergo surgery 1 week after interview (see Table 1).

**Table 1.** Participant demographics, interview length and whether they provided photographs.

Participant pseudonym and sex	Age	Time since diagnosis	Treatment status	Relationship and parental status	Interview length	Provided photographs
Hannah (female)	34	2 years 6 months	Post-treatment	Married O children	01-hour 58-minutes	Yes
Evelyn (female)	51	2 years 4 months	Post-treatment	Married 2 children	01-hour 21-minutes	Yes
Sofia (female)	32	1 year 5 months	Post-treatment	Married O children	02-hour 35-minutes	Yes
Faye (female)	55	4 years 6 months	Post-treatment	Married 2 children	01-hour 08-minutes	No
Gladys (female)	57	1 year 11 months	Post-treatment	Married 2 children	02-hour 33-minutes	Yes
Clara (female)	39	1 month	In-treatment	Married O children	02-hour 50-minutes	Yes
Tom (male)	30	3 years 4 months	Post-treatment	Married Expecting first child	01-hour 54-minutes	Yes
Mia (female)	26	2 months	Pre-treatment	Married 3 children	00-hour 50-minutes	No
Natalie (female)	46	3 months	In-treatment	Married O children	01-hour 46-minutes	Yes
Brian (male)	59	2 years 5 months	Post-treatment	Married 2 children	01-hour 17-minutes	No

### Materials

A study flyer, participant information sheet, photography guide, consent form, interview schedule and debriefing form were produced as part of this research and are available upon request.

### Data collection

Mesothelioma and asbestos related charities, research centres and support groups, based in the U.K., distributed the study flyer via online social media posts. In addition, Mesothelioma UK nurse specialists signposted the research to eligible patients. Individuals who expressed interest to take part were given a participant information sheet, photography guide and consent form by the study lead and provided the opportunity to ask questions before consenting to take part. After giving consent, an interview was scheduled for a date and time convenient to the participant.

Individuals who wanted to provide photographs as part of their experiential account could refer to the photography guide. This encouraged participants to take and share photos that they felt best captured their own personal experiences of living with mesothelioma, and this was repeated in communications with individuals to encourage them to tell their own stories with minimal researcher input and framing. However, individuals were asked to avoid taking identifying photographs of themselves or third parties, and any identifying pictures were edited using black censor bars in order to protect peoples’ identities. Individuals were asked to email photos to the researcher prior to interview so that they could be stored and discussed during the interview.

Online semi-structured interviews were conducted in 2021 and 2022. The interview schedule invited participants to introduce themselves, after which they were asked an open-ended orienting question: ‘*Generally speaking, what is living with mesothelioma like for you?*’. This allowed individuals to convey the salient aspects of their illness experiences that could be further explored throughout the interview. Subsequent questions went on to explore specific psychosocial elements of living with mesothelioma, including care and support. The schedule was used as a flexible guide and supplemented by follow-up questions in order to clarify and further expand on the participants’ telling of their own unique stories (Smith and Osborn, 2015). In cases where a participant had provided photographs, these were displayed on-screen using the ‘share screen’ function native to the platform and individuals were encouraged to discuss and reference these in the interview; including why they chose to share particular photographs, their intended meaning and why they felt it important to illustrate this.

The mean interview length was 1-hour 49-minutes, and it is worthy of comment that some interviews were especially long. This reflected a participant-led approach to the interview process, and the interviewer wanting to provide participants the space to convey their experiences in their own time; consequently, interview lengths varied according to the brevity or breadth individuals chose in sharing their accounts. Steps were also taken to respect participant’s time and energy, such as allowing individuals to take comfort breaks, as well as options to divide the interview into two or three more manageable sessions.

### Ethics

Ethical research procedures were implemented in accordance with the British Psychological Society’s: Code of Ethics and Conduct (2021b); Code of Human Research Ethics (2021a); and Ethics Guidelines for Internet-mediated Research (2021c), and approved by the host university faculty (reference: 412039).

### Data analysis

Interview recordings were transcribed verbatim, with photographs embedded within transcripts at the point of discussion. Text and photographic data were anonymised by removing identifiable information and substituting participant names for pseudonyms. Data analysis was informed by the six steps of IPA, described by Smith et al. (2022). Step 1: each transcript was read and re-read to help the researcher immerse themself within an individual’s account and consider salient and interesting aspects within it. Step 2: notes were made line-by-line in the left side margin to summarise descriptive, linguistic and conceptual issues related to the study. Step 3: notes and data were compared across the transcript in order to assess recurring similar and dissimilar concepts and identify ‘*personal experiential themes*’, that distil the core idiographic elements of a person’s account. Step 4: personal experiential themes were further abstracted to identify broader conceptual and interpretive links in an individual’s account. Step 5: the previous four steps were applied separately to each of the transcripts in order to produce an idiographic and in-depth case-by-case analysis of each individual’s lived experiences. Step 6: lastly, ‘*group experiential themes*’ were created through the careful cross-case comparison of transcripts and themes to identify peoples’ similar and dissimilar experiences of living with peritoneal mesothelioma. The verbal and visual data was analytically paired; with related text and photographic data consistently co-analysed and tabulated together, in order to privilege the participant’s own sense-making and voice when discussing their photographs and intended meaning. Therefore, analysis of photographs focused on the participant’s spoken thoughts and reflections of how an image illustrated or symbolised aspects of their lived experiences.

In line with good IPA practice, this analysis sought to balance the interpretation of what is shared across multiple cases while recognising an individuals’ distinct and divergent experiences (Smith et al., 2022). The analysis also sought to evidence analytical claims within the verbatim text of participants accounts, and ‘member-checking’ was used – allowing individuals to state whether personal experiential themes reflected their experiences. Member checking was done individually: with each participant able to give feedback on the analysis of their own interview, as a separate case study, to help support an idiographic approach to validation and rigour (Smith et al., 2022). Three participants did not provide any feedback. All other participants did provide feedback to say themes reflected their experiences and views shared in the interview.

### Reflexivity

IPA researchers engage in a double hermeneutic process when trying to make sense of the participants’ sense making, and this invites consideration of the researchers’ role and influence when collecting and analysing the data (Smith et al., 2022). This reflexivity is thus provided to help readers critically consider and contextualise the current research and findings below.

The first author conducted this research as part of their PhD with the input and support of co-authors (PhD supervisors). Although the first author had a great-aunt who died of pleural mesothelioma in 2019, authors otherwise had little prior personal knowledge or awareness of mesothelioma. In addition, the first author (a man in his early 30s) conducted the interviews and was several years younger, and of different sex, to most participants. This positions the research team as both experiential and demographic outsiders, and could have unknowingly influenced the research process; for example, participants may have been more self-conscious and less willing to discuss certain topics with a person they viewed as less relatable or understanding. That said, the length of most interviews as well as the depth and frank nature of data suggests that this was not typically the case.

Online interviewing can also present technical issues and limit the researcher’s ability to foster rapport. However, no major technical issues were encountered by the researcher or reported by participants, and the interviewer used clarifying questions, non-verbal gestures, and made encouraging remarks to help convey sincere interest and to reassure individuals that their voices were being heard (Seitz, 2016).

## Results

**Figure 1.**
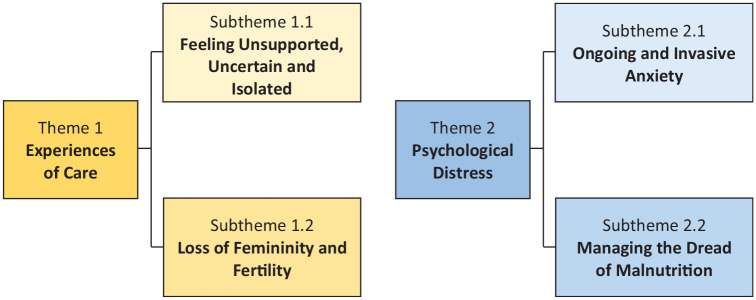
Thematic map illustrating two themes and subthemes of living with peritoneal mesothelioma.

Two themes that each have two subthemes are presented in this paper: (1) ‘Experiences of Care’; and (2) ‘Psychological Distress’. In keeping with our aim and phenomenological practice more generally, these themes explore the more unique experiences and anxieties shouldered by individuals living with the peritoneal variant of mesothelioma, that have yet to be identified in the wider research literature. Both themes and their respective subthemes are represented via thematic map (see Figure 1), and are outlined in detail alongside relevant extracts and photographs in order to illustrate individuals’ lived experiences.

### Theme 1: Experiences of Care

This theme explores individuals’ experiences of clinical care and support for peritoneal mesothelioma, highlighting the ways that participants felt initial support was lacking, with female participants voicing additional unmet needs and concerns. Two subthemes are presented:

○ Subtheme 1.1: Feeling Unsupported, Uncertain and Isolated
○ Subtheme 1.2: Loss of Femininity and Fertility

#### Subtheme 1.1: Feeling Unsupported, Uncertain and Isolated

Reflecting on their early care experiences individuals often described a long difficult diagnostic journey, wherein clinical symptoms were regularly misdiagnosed as cancer of the ovaries, bowels or appendix, culminating in the need for multiple appointments and tests, over a period of several weeks or months. For some, the scarcity of answers and apparent medical speculation and misdiagnosis, elicited feelings of increased anxiety and frustration:

*So with this* [diagnostic] *rollercoaster that I said to you about: one minute thinking, I’ve got like leukaemia and then I’ve got ovarian that spread – that’s really bad. And I was still googling things at that point, which is really bad.* **(Clara)**

*They just said, “it’s cancer”, and I said, “well, where’s the cancer?”* [They said] *“I don’t know, it might be your stomach.” So then, obviously, I immediately go on Google and start researching stomach cancer and then I’m researching bowel cancer.* […] *Why didn’t they just ask me in for another biopsy if they didn’t know? It’d have been better if they didn’t tell me anything until they knew because all it did were make me, at that point, really worry sick.* **(Natalie)**

Like Clara and Natalie, some individuals were upset by medical speculation and felt this only served to worsen an already stressful juncture in the care pathway, as they began to fixate on the potential cause of symptoms and seek answers online. Upon diagnosis, multiple participants continued to describe an uncertain care pathway as clinicians appeared unfamiliar with peritoneal mesothelioma and unsure of what advice to give:

*I think the consultant didn’t know really where- which direction I was going, and he knew he could refer me on but he didn’t have any answers for me. So, at that stage I was given diagnosis and for about a week or so I didn’t have any further information* **(Evelyn)**

[Gynaecologist] *said, “It’s peritoneal mesothelioma.” And I said, “Oh, what’s that?” And she just said, “Oh, here’s a leaflet, we don’t really know about it. It’s not really anything to do with gynaecology, but here’s a leaflet.* **(Sofia)**

Here then we begin to see the potential shortcomings of early clinical care which appeared to leave a number of people isolated in their diagnosis and unsure of where to go for additional medical support. Moreover, these accounts convey a sense that participants moved from the fear and frustration of not having a diagnosis to the isolation of not knowing what support to access post-diagnosis. This sense of inadequate support and isolation early in the peritoneal mesothelioma journey was further contrasted by some to pleural mesothelioma – which was perceived to be better supported:

[the clinician] *said that* [peritoneal mesothelioma] *is actually even more rare than the* [pleural] *one but obviously, there’s such little research into this one.* **(Mia)**

*It is isolating because I think a lot of people search the Facebook support groups for someone in a situation like them* […] *And when you mention it, first of all, people don’t know what it is, but then if you say, “Oh, it’s the cancer related to asbestos*^1^*”, they’re like, “Oh, is it not something that old men get?” So, yes, you are sort of isolated for a number of reasons. And, yes, I think the fact that the support groups do cater mostly for pleural patients, it does further isolate people.* **(Hannah)**

For these individuals, peritoneal mesothelioma may be more isolating due to the wider lack of research and awareness around the condition. Hannah’s quote is especially illustrative of this as she recalls the difficulty of finding a support group for peritoneal mesothelioma; explaining that most support groups are aimed at people with pleural mesothelioma. In this way, those living with peritoneal mesothelioma risk feeling unseen and unsupported in an illness space that is commonly characterised by pleural cases and older men.

In summary, this subtheme explores individuals’ difficulties getting an accurate diagnosis and accessing subsequent support. For many, the diagnostic journey was plagued by multiple tests and misdiagnoses, which increased feelings of anxiety and frustration, with some individuals further criticising the lack of follow-on information and signposting after diagnosis. Indeed, some individuals articulated a sense of isolation living with peritoneal mesothelioma due to the lack of support, and general lack of awareness among the public and healthcare body – especially compared to pleural mesothelioma.

#### Subtheme 1.2: Loss of Femininity and Fertility

Further to the lack of initial support and signposting discussed in the previous subtheme, a number of female participants reported additional issues and treatment sequalae for which there was little available support. These concerns were not evident in the accounts of male individuals, and suggests a gendered element in how women are uniquely affected – with one such issue regarding the impact on body image:

*I took it* [photograph] *because like frustrated that day. Like trying to look and just be like, oh my stomach looks massive. I just kind of go in and out of that: like, oh it’s not that bad one day, and just try not to concentrate on it too much. But then there’s, I think the day that I took that, it was like annoying me, and there’s nothing I can do.* **(Clara)**

*I’m thinking of getting like a tattoo, so I don’t look at it, I’m not reminded of it* [scar] *to kind of just cover it up so, because I’m quite conscious of it a wee bit sometimes like so when I’ve got yoga, I’m always wearing big baggy t-shirts.* **(Sofia)**

**Photograph 1.**
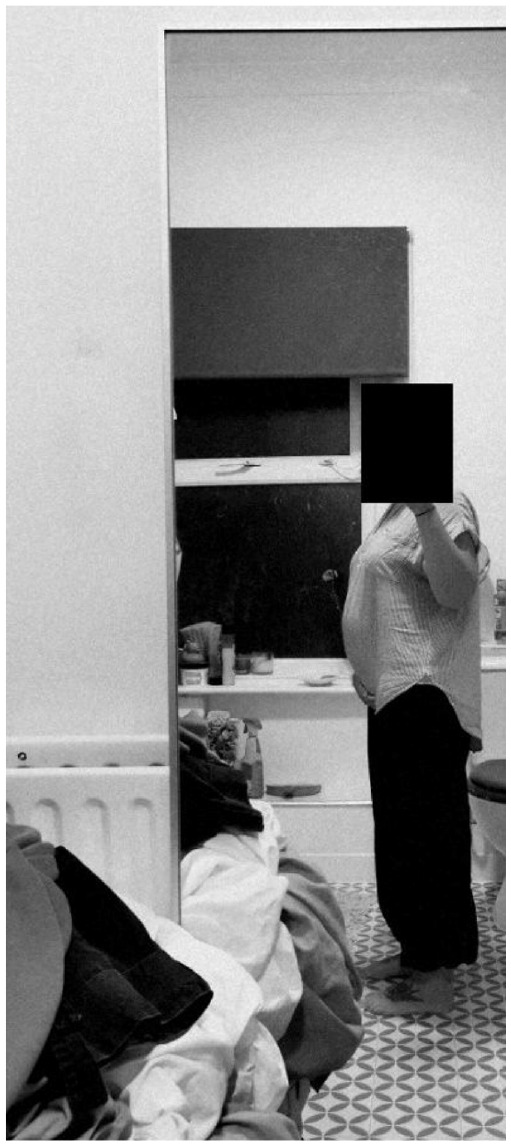
By Clara.

**Photograph 2.**
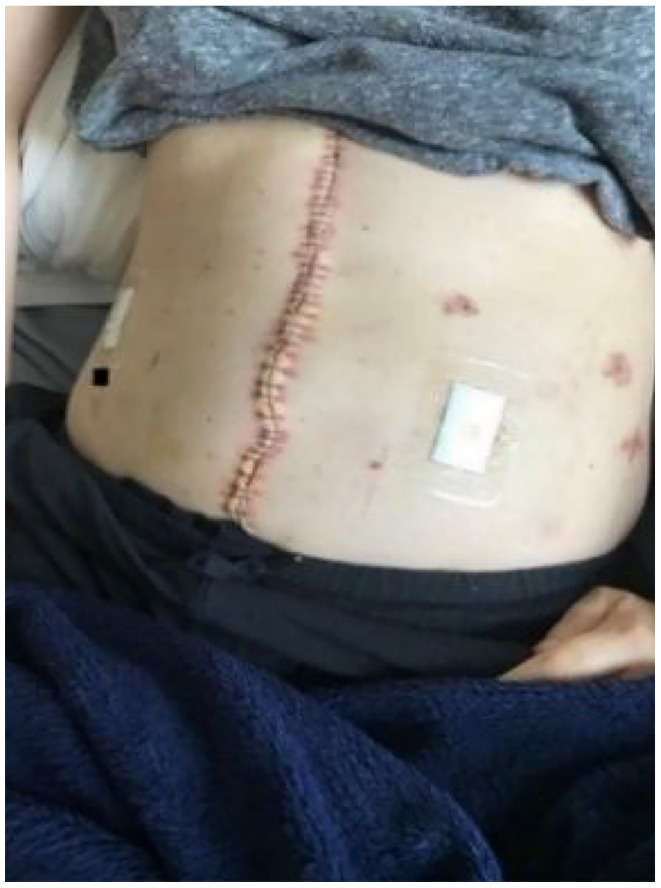
By Sofia.

Clara and Sofia photo and discuss the perceived adverse effect peritoneal mesothelioma had on their appearance, either as the result of abdominal swelling pre-surgery or abdominal scarring after surgery; and which elicited feelings of frustration or embarrassment. Moreover, by comparing the two extracts, it may be possible to observe how negative body image presents differently over the illness experience: either as concern about symptomatic swelling before treatment or abdominal scarring after treatment. Some individuals also felt unsupported in their efforts to manage the physical and psychological impact of medically induced menopause that can form part of the treatment pathway:

*The things that I have been worrying about. About the way in which my body has been ageing, which, you know, the little person in the back of your mind is saying, “It’s cancer, it’s cancer, it’s cancer—it’s come back!”* […] *It’s because, like I said to that nurse, “Should I be speaking to somebody now about H.R.T.* [hormone replacement therapy]*?” And she boohooed* [dismissed] *me.* **(Gladys)**

*The changes it makes in your body afterwards, I mean on a sort of sexual relationship level, it’s not really the same as it was before and it’s basically because your body has changed. So, I think that’s quite a significant challenge and I think in general there’s a lack of support out there for things like that.* **(Hannah)**

Gladys and Hannah both describe adverse sequalae after undergoing medically induced menopause, in terms of perceived ageing and impaired sexual ability, with neither feeling adequately supported to manage these effects. Moreover, Gladys’ repetition of ‘*its cancer*’ indicates significant alarm and the strength of this intrusive and upsetting thought which is responded to by a nurse who ‘*boohooed*’ her fears. This conveys a sense of profound distress that was met with indifference, thus leaving Gladys with a sense of her illness being invalidated and trivialised. Indeed, the word *boohooed* suggests the nurse is actually scornful of Gladys’ suggestion. These extracts thus provide insight of potentially broader psychosocial concerns that relate to clinical treatment: with Gladys citing fears of cancer recurrence and Hannah experiencing diminished sexual intimacy. Another issue addressed the inability to conceive and have children:

[I’m] *thinking, oh God, I might lose me ovaries now might never be able to have another child. I’ve already got three boys, but I’ve always wanted that little girl, do you know what I mean? And I’m young still.* **(Mia)**

*I can’t have children and that’s a big thing so. And I’m now menopausal as well; I’m in the menopause and that’s a big thing.* **(Sofia)**

*You’ve got to get used to the fact that you just lost all your childbearing ability. And does that make you, I don’t know, less of a woman? It’s quite hard and everyone’s kind of matter-of-fact about it, and you’re thinking actually it’s changed me.* **(Faye)**

The loss of their childbearing ability presented an additional worry for a number of female individuals. For Mia aged 26, and Sofia aged 32, this worry appeared to intersect with their age, menopausal status and sudden loss of reproductive ability; with Mia lamenting, ‘*I’m young still*’, and Sofia acknowledging, ‘*that’s a big thing*’. This is discussed further by Faye in view of more traditional notions of womanhood which may be impacted by the loss of childbearing ability, resulting in significant psychological distress for which Faye felt she had little support.

In summary, this subtheme explores the distinctive concerns reported exclusively by female individuals which included negative body image, perceived ageing, diminished sexual function and loss of fertility. Most issues developed as the result of vital life-extending treatment, often due to induced menopause; with individuals struggling to manage the physical, psychological and gendered concerns which stem from surgical scaring, rapid hormone change and inability to have children.

### Theme 2: Psychological Distress

This theme explores individuals’ broader psychological experiences and coping strategies in living with peritoneal mesothelioma, highlighting acute feelings of anxiety, as well as chronic fears of malnutrition and sense of coping through dietary practices. Two subthemes are presented:

○ Subtheme 2.1: Ongoing and Invasive Anxiety
○ Subtheme 2.2: Managing the Dread of Malnutrition

#### Subtheme 2.1: Ongoing and Invasive Anxiety

Reflecting on their broader experiences, every individual voiced in great detail the profoundly negative impact living with peritoneal mesothelioma had on their mental health, which started for many at the point of diagnosis:

*I went into* […] *meeting with the consultant that give me my diagnosis, absolutely bricking it* [scared]. *And then he did say, “oh it, it shows mesothelioma”. And I pretty much started crying with my mum.* **(Mia)**

*I can remember I was sitting in the chair; the professor was off to my right and the meso nurse was sitting on one of those beds* […] *And when they started to explain to me what it was, and the enormity of it, it was that, that tunnel vision came in and everything closed down* […] *and I know what it is – and it’s your whole world shrinking down.* **(Brian)**

Mia and Brian both report how deeply upsetting it was to be diagnosed with peritoneal mesothelioma. Brian describes his ‘*whole world shrinking down*’ – evoking a powerful sense of his entire lifeworld collapsing in that moment, as well as feelings of palpable, existential shock that were evident in the accounts of other people. More generally, after diagnosis individuals described poignant feelings of acute anxiety, scanxiety and post-traumatic stress; indeed, these feelings were common, and appeared to permeate and define the immense psychological burden living with peritoneal mesothelioma:

*I have found it mentally more traumatic. The physical aspects have been easier to deal with than the mental trauma… huge anxiety, huge anxiety, yes, massive, yeah.* **(Gladys)**

*With the scans, I’m anxious every year that one of the scans they’re gonna say, “Oh my God, this has changed. We’re gonna have to do this now”. It’s not one of those where it kind of resolves and they can just take it away.* **(Tom)**

*It was anxiety with a small amount of post-traumatic stress that I would say. Like I would have like flashbacks to like the surgery, have nightmares that I’d have to go through it again. So, there was an element of like post-traumatic stress, but the majority was anxiety. Feeling overwhelmed, that I just couldn’t cope with things, and very emotional.* **(Hannah)**

For some, anxiety further manifested via intrusive thoughts and disturbed dreams that impaired sleep:

*The sleep one* [photograph] *is about in waking up in the night and the worst thoughts pop-up. I have had a lot of sleep issues really* […] *one of the greatest impacts.* […] *I’ve really struggled, obviously the first diagnosis to sleep, there were long periods when I’d be awake* **(Evelyn)**

*Sometimes you forget and I’m carrying on* […] *And then when it hits you -it hits you again- and I’m having very vivid dreams all since I’ve been diagnosed. It’s weird, vivid dreams, weird dreams, and then I wake up and when you realise -you forget initially- and then you remember and think, oh no, it hits you like a ton of bricks.* **(Natalie)**

**Photograph 3.**
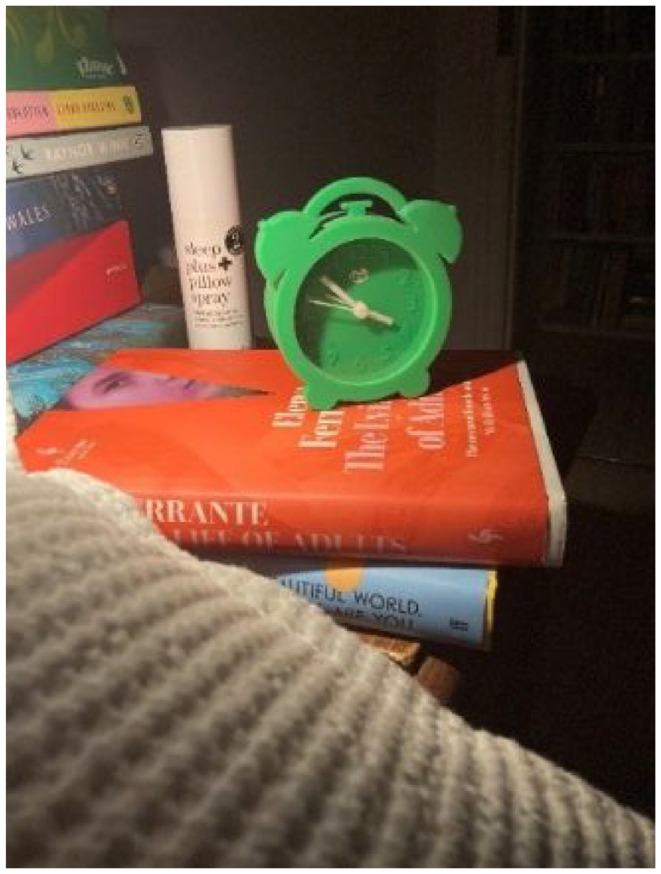
By Evelyn.

Together, the extracts capture the acute psychological distress of living with peritoneal mesothelioma, via the powerful use of metaphor (‘*ton of bricks*’) and extreme language (‘*the worst thoughts*’) to convey the enormity of the psychological impact. Although most individuals at the time of interview had been cancer free for several months or years, following treatment of the initial tumour, they still reported continued feelings of anxiety and concern at the thought of the cancer returning. This was depicted by some as a ‘*niggle*’ in the back of the mind that they tried to not think about – as they chose instead to ‘*compartmentalise*’ or suppress this fear:

*Quite often I’m having a little mental battle within myself about how much headspace it takes up when you don’t want it to. So, you have to kind of box it up and put it to one side and then just get on with everything.* **(Faye)**

*I have to sort of like, suppress that* [anxiety] *and put that to the back of my mind.* […] *it’s a constant barrage of people dying of cancer and me sitting there thinking, yeah and, and that is going to be me at some stage. And it’s those sorts of things that I have to, if I, if I have the mental strength to push back to the back of my mind.* **(Brian)**

Faye and Brian echo the views shared by others who similarly discussed feelings of long-term anxiety, and desire to manage this via distraction or suppressive strategies; as individuals recalled keeping busy so as to avoid negative thoughts and emotions, and focus on more positive aspects of their daily living, such as: learning new hobbies, focus on family and/or other meaningful outlets. However, like others, Faye and Brian reflect on this as an ongoing process: as a ‘*mental battle*’ that takes ‘*mental strength*’. This suggests that those living with peritoneal mesothelioma may shoulder acute and chronic anxieties that require significant sustained effort to cope with.

In summary, this subtheme explores the acute and chronic anxiety bore by every participant following the diagnosis of peritoneal mesothelioma. Individuals described vivid and pervasive feelings of anxiety, scanxiety and post-traumatic stress that could significantly disturb sleep, and that participants tried to manage via strategies to distract or suppress thinking about.

### Subtheme 2.2: Managing the Dread of Malnutrition

Further to the broader anxieties explored in the previous subtheme, a number of individuals discussed more specific fears related to cancer recurrence. Notably because peritoneal mesothelioma affects the abdomen and local gastric organs – like the stomach and colon – these fears often fixated in how possible future tumours could harm a person’s ability to digest food:

*I think about the future and how, I mean, essentially think what it might be involved starving to death really if it does, the impact of peritoneal being that you just can’t get enough nutrition in. That does frighten me*. **(Evelyn)**

*So you’ve got all this* [anxiety] *going round in your mind like, is it going to grow? Am I going to need another operation? Oh my God, is it going to stop my bowels working? Is it going to stop my stomach working? So that does swim around the whole time*. **(Faye)**

Both extracts serve to illustrate the more unique anxieties felt by those with peritoneal mesothelioma; with Evelyn and Faye voicing chronic fear about the possible impact of future tumours on their abilities to digest food – thus eliciting fears of death due to severe malnourishment and starvation. In response, some individuals recalled changing their diets in part to reduce abdominal discomfort and constipation when eating food following cytoreductive surgery. Additionally, diet modification appeared to enhance an individual’s sense of psychological coping:

*I just thought if I do this juice diet* […] *then that will just take my mind off it because they did the cancer, for two weeks, the cancer pathway thing. So, for a couple of weeks, I just followed that diet religiously. And it took my mind off of everything.* **(Clara)**

*I want to put on that extra bit of weight. It is security to me. It gives me a little bit more time, buys me a little bit more time.* **(Brian)**

*I don’t waste my time eating food that I think is below par, you know. And I think that’s like a protective mechanism for myself.* […] *Because those are things that are completely within my control, aren’t they? What I put into my body and how I use my body.* **(Gladys)**

**Photograph 4.**
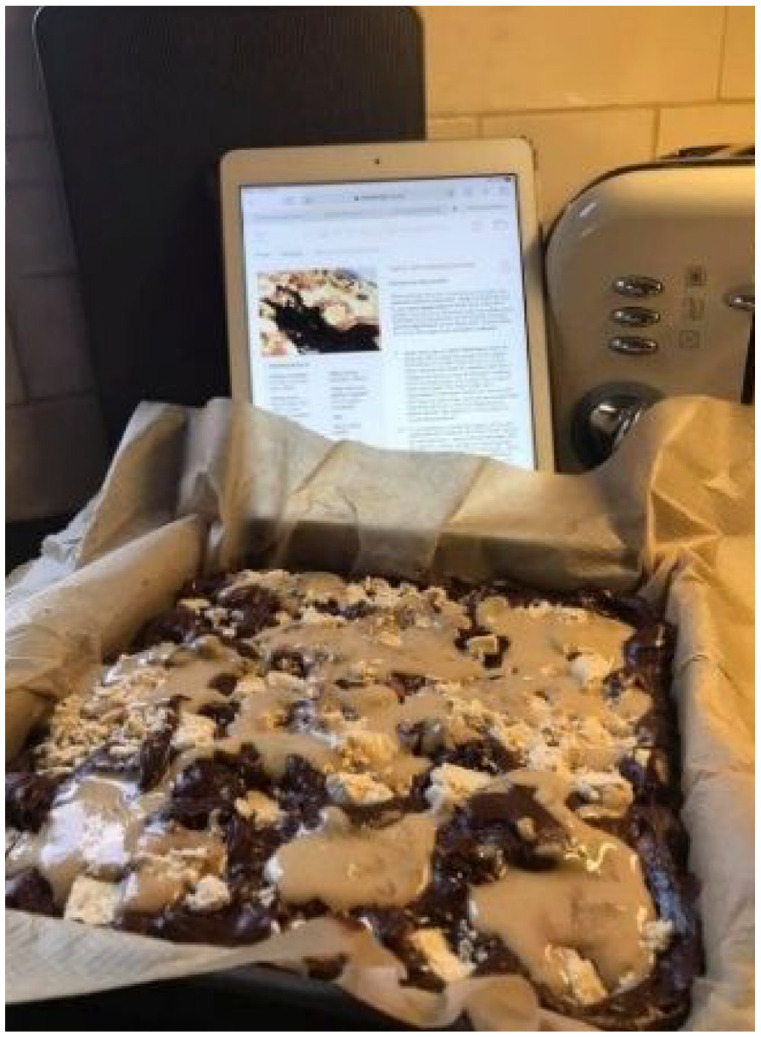
By Gladys.

Clara, Brian and Gladys all credit diet modification for supporting their sense of coping and well-being, though this did vary between participants. For Clara, adopting a juice-based diet helped to occupy her, possibly indicating that a strict (‘religious’) focus on diet helped redirect her attention away from more negative thoughts or feelings. Brian and Gladys however put more emphasis on increasing body weight to reduce risk of starvation if the cancer returns; Brian views weight gain as a ‘*security*’ that offers him ‘*a little bit more time*’, and Gladys sees it as a ‘*protective mechanism*’ depicted via calorie dense cake. Notably, Gladys’ image of the gooey sumptuous cake is evocative of typically unhealthy foods and diet, and may be a deliberate choice be her to redefine what healthy eating means: to prioritise weight gain, before weight loss. Moreover, the use of security and protective language by Brian and Gladys suggests that increasing body weight may offer a sense of health security that they otherwise feel is threatened.

These accounts thus help to reveal the potential psychological value of dietary practices to people with peritoneal mesothelioma as a coping mechanism; whether as a means to avoid negative thoughts and feelings, or as a means to engender feelings of security and control over their health. Such perspectives and means of coping were however disputed by other participants:

*To be honest there’s no actual evidence that it does stop cancer from returning. So, yeah, you do hear things from people. But there’s a lot of false information and I think that’s part of the problem. You do hear from people that have changed their diet because they don’t want cancer to return but in fact, like, they could actually be doing more harm than good.* **(Hannah)**

While acknowledging individual’s motivations to adapt their diets to manage the risks posed by cancer, Hannah warns of potential misinformation among peritoneal patients; stating that some may be at risk of harm due to incorrect dietary advice. Consequently, there is a need to recognise the possible benefit and risks of dietary practices; indeed, diet modification might help individuals to ease abdominal pains, and engender feelings of security and control which in turn ease feelings of chronic anxiety and threat. However, in the absence of nutritional and oncological guidance, the uptake of certain dietary practices may be misinformed – rooted in unrealistic expectations and false hopes – and possibly even present a substantive risk to health.

In summary, this subtheme explores fears of cancer recurrence and impact on abdominal functioning, in terms of malnourishment. In response, several individuals credited diet modification as a means to help alleviate physical symptoms and as a means to enhance their psychological coping and well-being. However, some further warned against the prevalence of diet-based misinformation amongst patients. This therefore merits a more nuanced understanding of dietary practices and peritoneal mesothelioma which recognises people’s desires to manage their health and anxiety but also avoids the perpetuation of misinformation.

## Discussion

This is the first paper to use a phenomenological approach to study the accounts of people living with peritoneal mesothelioma; and provides a novel understanding of their care experiences, anxieties and coping strategies, thus setting it apart from other research which has mainly focused on the psychology of individuals with pleural mesothelioma (Lond et al., 2022; Sherborne et al., 2024).

Regarding care and support, although individuals were very appreciative of the medical care received, most experienced a lengthy diagnostic journey that was beset by clinical speculation and misdiagnosis, which caused feelings of uncertainty and anxiety. This reflects the experiences of individuals living with rare diseases more generally; wherein absent, incorrect and/or delayed diagnoses can elicit feelings of pronounced uncertainty, anxiety, frustration, loneliness and fear (Llubes-Arrià et al., 2022). Moreover, recent survey findings reveal that while 87% of peritoneal patients were satisfied with their treatment, 45% perceived avoidable delays in diagnosis (Ejegi-Memeh et al., 2024). Indeed, the condition remains a diagnostic challenge because it typically presents with non-specific symptoms which delay diagnosis and subsequent access to treatment (Razzak et al., 2022; Vidal et al., 2022).

Even after being diagnosed, multiple individuals continued to find it hard to locate and access support. This echoes the concern of pleural mesothelioma patients who similarly find it difficult to navigate care (Clayson et al., 2005; Kasai and Hino, 2018; Lee et al., 2009). However, – unlike their pleural counterparts – individuals in this study also felt there was a lack of knowledge and awareness around peritoneal cases, both among clinicians and the general public; whose awareness of peritoneal mesothelioma appeared to be limited and overshadowed by lay perceptions of (pleural) mesothelioma as an ‘*old man’s disease*’. In addition, some female individuals felt there was little help to manage the physical and psychological sequalae of early onset menopause, which can have a long-term negative impact on cognition, mood and sexual health (Faubion et al., 2015). In this way, we see how people with peritoneal mesothelioma, and women in particular, risk feeling overlooked within a healthcare system and illness space generally associated with older individuals living with pleural mesothelioma. This reflects a broader lack of funds and research into women’s health conditions, like: endometriosis, chronic fatigue syndrome and multiple sclerosis (Mirin, 2021), and renders those women living with these conditions comparatively understudied and unsupported relative to their male peers.

Regarding the psychological effects of living with peritoneal mesothelioma, individuals voiced in detail acute feelings of anxiety, scanxiety and post-traumatic stress that could persist for many months/years which manifest as fears of cancer recurrence. This echoes the anxiety of pleural mesothelioma patients (Arber and Spencer, 2013; Clayson et al., 2005; Kasai and Hino, 2018; Lee et al., 2009; Taylor et al., 2019; Walker et al., 2019), and higher incidence of anxiety in cancer patients generally (Mitchell et al., 2013). In contrast, while individuals with pleural mesothelioma may fear dying due to the build-up of fluid in the lungs (Clayson et al., 2005; Walker et al., 2019), some in this study feared dying due to malnutrition and sought to avoid this via diet modification which seemed to engender a sense of coping and control. However, as acknowledged by some participants, the wide availability of online dietary misinformation for cancer risks individuals adopting unhealthy and even dangerous food practices (Warner et al., 2022). Indeed, the current analysis suggests that peritoneal patients may be at a higher risk of misinformation since the cancer affects the abdomen and adjacent digestive organs.

### Implications for practice

There is need to increase and better signpost services throughout the healthcare pathway and beyond, to help peritoneal patients feel less isolated and more confident in their medical journeys. In addition, targeted support for those undergoing medically induced menopause need to be developed to address concerns about body image, sexual intimacy and fertility. Findings also demonstrate the need to help individuals manage feelings of anxiety and post-traumatic stress, as well as fears of cancer recurrence. To this end, cognitive-behavioural interventions aimed at increasing individual’s perceived confidence to cope in the event of possible recurrence, and further use activities that promote mindful distraction and cognitive flow may help patients deal with feelings of anxiety (Derry-Vick et al., 2023).

The provision of expert dietary guidance should also be considered, in view of individuals altering their diets in order to manage the physical and psychological burden of living with peritoneal mesothelioma. Research finds that mesothelioma patients generally may employ dietary practices to foster a sense of psychological coping and control (Arber and Spencer, 2013; Clayson et al., 2005; Walker et al., 2019), however this appears to be especially salient for those with peritoneal mesothelioma due to its impact on the digestive tract. Evidence based resources are thus warranted to help individuals understand the role of nutrition in living with mesothelioma; and empower them to make more informed food choices that engender a sense of proactive coping and well-being.

### Strengths and limitations

This study is the first to provide a detailed account of how people experience peritoneal mesothelioma. The use of in-depth interviews in combination with participant generated photographs and individual member checking aided the deep and idiographic exploration of each person’s account, in line with an IPA approach that favours the study of peoples’ unique journeys in lieu of making broad generalisations (Smith et al., 2022). In doing so, this study highlights several issues which may affect peritoneal patients that had previously been unexplored in the literature. That said, the sheer volume of experiential data from across the participants’ accounts cannot be done analytical justice within the scope of this report. We therefore invite readers to read the associated themes tables (Supplemental Table 1), for a better understanding of how individuals reflected on the issues discussed.

It should also be noted that all participants who chose to take part were: White, heterosexual, married and mainly women (8/10 participants), and had received or were going to receive cytoreductive surgery and intraperitoneal chemotherapy – though Clara’s clinical pathway was uncertain at the time of study. As a result, the current data and analysis is likely to better reflect the perspectives of such individuals, which leaves the voices of people who are non-White, non-heterosexual, single, male and/or receiving other forms of treatment comparatively unheard or explored. That said, in regard to the current paper, having a more diverse sample may have challenged the relative homogeneity required for IPA research to be relevant and meaningful when exploring the experiences of particular groups (Smith et al., 2022). Conversely, the range in participant age and time since diagnosis limits the homogeneity of our sample, though this is partly offset by the aforementioned similarities.

### Future research

Although the findings highlight several issues that may negatively affect patients, including feelings of isolation, anxiety and concern regarding body image, sexual intimacy and fertility, and dietary change, future research is needed to survey the prevalence of these issues in the wider patient population to allow for more generalisable conclusions which support the development of targeted resources to help this patient demographic. Future research should also study the accounts of more diverse samples to broaden our understanding of the illness experience in relation to peoples’ backgrounds.

## Conclusion

This paper aimed to explore the concerns and support needs of people with peritoneal mesothelioma, to address extant research gaps and guide the development of therapeutic interventions and practices. By applying an in-depth phenomenological approach to the collection and analysis of experiential data, we present novel insights which underline the need to increase supportive care for peritoneal patients, who otherwise risk feeling unsupported and unseen, and who shoulder chronic feelings of anxiety and fears of cancer recurrence. Services are therefore warranted to help individuals cope with the sequalae of medically induced menopause and acute distress of living with peritoneal mesothelioma in general, as well as help individuals modify their diets in support of their physical and psychological well-being.

## Supplemental Material

sj-docx-1-hpq-10.1177_13591053241298932 – Supplemental material for The psychological impact of living with peritoneal mesothelioma: An interpretative phenomenological analysis Supplemental material, sj-docx-1-hpq-10.1177_13591053241298932 for The psychological impact of living with peritoneal mesothelioma: An interpretative phenomenological analysis by Benjamin Lond, Lindsay Apps, Kerry Quincey and Iain Williamson in Journal of Health Psychology

## Notes

1. Pleural mesothelioma is caused by the inhalation of asbestos fibres several years before the onset of disease.
