# Supplementary material for: The psychological impact of living with peritoneal mesothelioma: An Interpretative Phenomenological Analysis
Source: J Health Psychol. 2024 Nov 25;30(10):2781–97. doi: 10.1177/13591053241298932 (PMC12381386; doi:10.1177/13591053241298932)
Supplement: sj-docx-1-hpq-10.1177_13591053241298932 – Supplemental material for The psychological impact of living with peritoneal mesothelioma: An interpretative phenomenological analysis [file sj-docx-1-hpq-10.1177_13591053241298932.docx]

**Supplemental Material**

Supporting quotations for both themes and subthemes are tabulated below. Table colours correspond andw align with colours in the Figure 1 thematic map presented in the online article: yellow = Theme 1, and respective subthemes, and blue = Theme 2 and respective subthemes.

| Peritoneal Mesothelioma Themes Table | | | |
| --- | --- | --- | --- |
| Theme | Subtheme | Participant | Example Quotations |
| Theme 1  **Experiences of Care** | Subtheme 1.1 **Feeling Unsupported, Uncertain and Isolated** | Hannah | I actually go to, belonging to another support group, which is just for people who’ve had cancer in their 20s, 30s and 40s, and they all report the same. That in the breast cancer clinics the majority are in their 60s. You get someone in there who is in their 20s and, you know, they feel a bit isolated. And that seems to a common theme with everything, not, not just … Yes, breast cancer, bowel cancer, they tend to be, you know, the older generations that have it, and actually, yes, including the younger generations is a bit difficult. |
|  |  |  | I think it is isolating, because I think that a lot of people search the Facebook support groups for someone in a situation like them, because they’ve not seen it before. And when you mention it […] First of all, people don’t know what it is, but then if you say, “Oh, it’s the cancer related to asbestos,” they’re like, “Oh, is it not something that old men get?” So, yes, you are sort of isolated for a number of reasons. And, yes, I think the fact that the support groups do cater mostly for pleural patients, it does further isolate people. |
|  |  |  | I think as well, when you look at the statistics, I think most of the statistics are for your average patient that’s in their 70s or 80s, probably got pleural… which is a lot different to my situation. I mean it would be nice to see figures for those that have had debulking surgery and seeing what the survival rates are then. Because I think it’s, it’s a bit unfair really to judge us all in the same group, when we’ve got totally different circumstances. |
|  |  |  | And I think […] getting the image out there that peritoneal mesothelioma is different and carries with it a separate set of statistics. Because, yes, I don’t think you can sort of compare them all together. |
|  |  |  | I did go to this Manchester Asbestos Group like support sessions, but I found that the vast majority were in their 80s, and it was all very focused towards pleural. So, one week they had a nurse come in to show them how to drain a pleural drain, how to empty it, which obviously is not going to apply to me. There was also an app that they developed so that patients could record symptoms, but again all the symptoms were to do with like shortness of breath and things like that, that the average peritoneal patient is not going to have. Ours is more likely to be digestive discomfort, bloating, swelling, that sort of thing. So, yes, I mean I know that the majority of cases are pleural and not peritoneal but, yes, it does seem very set up for pleural. |
|  |  |  | And even now like I’ve had to stop going to the support meetings because they’re always done during the day. Like Monday at like 11 o’clock, which are great, it’s great if you are retired and you’ve got pleural, but if you work full time then it’s impossible for me to go, where evenings would work better for me because, you know, or a weekend or something. But, yes, I feel that everything is catered towards pleural. |
|  |  |  | So, I suggested to Mesothelioma UK that they actually update their website, because they had huge, huge sections on pleural and absolutely nothing about peritoneal. Just a specialist nurse called Sam, and that was literally it. I said to them, “Well, you know, for pleural you’ve got all treatment options, clinical trials, everything, but there’s nothing about that, or even explaining what the peritoneum is,” because it just doesn’t explain anything at all. |
|  |  |  | But, yes, I sort of felt that there wasn’t support there, and that the support groups on Facebook, that I belong to, is people desperate for information because they can’t find information anywhere else. |
|  |  |  | There is one that’s peritoneal specific, but a lot of them are pleural. […] but again a lot of the posts are related to pleural rather than peritoneal. And the thing is that the patients … I mean some may have similarities in turn, but in general, you know, you find that the peritoneal patients are younger and they’re more likely to be female, where pleural is most likely to be male. And, yes, I guess it, you know, it’s two completely different groups. |
|  |  |  | And they said that Basingstoke had retested the biopsy and I had a high-grade cancer and not a low one as we’d originally thought. But that they were inviting me down for a consultation to Basingstoke. So, I had a lot of questions for my oncologist at this point, mostly about […] Why did Manchester think it was low-grade and then it was retested, and they said it was higher grade? And my oncologist could not answer these questions. |
|  |  |  | I had to wait three weeks for the results, and they came back inconclusive. So, I had to have a second laparoscopy. So that’s another three, four weeks waiting for that one until we got the results back. So, I think it was June when I got the diagnosis. So, it was a lot of waiting around. […] So, there was a lot of waiting around and, yes, a lot of time off work, because each time I had the laparoscopy I was then off sick from work, because obviously I couldn’t, I couldn’t work. So, yes, there was a, it was a long year. A lot of waiting around. And, yes, it wasn’t a great time. |
|  |  | Evelyn | So at that point [diagnosis] I think the consultant didn’t know really where, which direction I was going and he knew he could refer me on but he didn’t have any answers for me. So at that stage I was given diagnosis and for about a week or so I didn’t have any further information |
|  |  |  | So that period of limbo I think was perhaps the worst stage not knowing who was going to treat me and how it was going to be approached. |
|  |  |  | And there is that sort of tendency I think you want to know facts and figures that, that what is the prognosis, what, how long are you going to have and those sorts of things. And they are perhaps difficult to find but they’re not necessarily things that a medical establishment would want you to have anyway because they’re just, you’re an individual and you’re not necessarily going to follow that pattern. |
|  |  |  | there’s this sort of, that idea of looking to the future and wanting to know. But of course, you, they can’t, can’t, yeah, no, can’t keep informing you as an individual. |
|  |  | Sofia | And I said, ‘Oh, what’s that?’ And she just said, ‘Oh, here’s a leaflet, we don’t really know about it, it’s not really anything to do with gynaecological, but here’s a leaflet.’ |
|  |  |  | the team based here are kind of predominantly pleural so at the beginning like their knowledge wasn’t as known. So I was getting referred to Basingstoke but you only really got referred to Basingstoke like if you were really getting the surgery. |
|  |  |  | I was a bit like, ‘What happens? What about other stuff? What is the clinical trials for peritoneal?’ And it was a bit like, ‘Oh, we don’t really know.’ And I had this like paranoia being like, ‘Oh, if I don’t get the surgery what else have I got? I need, I need to be told.’ So I think that maybe more of a focus on the peritoneal. |
|  |  |  | I think they need to put like positive case stories on that from a peritoneal point of view because you have the stories, the positive stories from pleural but there’s not, there’s lacking in peritoneal. And they kind of just then just like signposted Basingstoke and the Basingstoke website is not the best either […] that’s why I ended up like scouring on Instagram |
|  |  |  | So I think that maybe more of a focus on the peritoneal. Or even if there was an explanation to say like, say if we were doing like a, a study for pleural it would probably help peritoneal like it’s one of those things that it’s like a lot of the drugs now that they’re finding help pleural is helping peritoneal. So even if they explained it that way then … I just think at the beginning there could be more like information given. |
|  |  |  | I found these ladies that are like been diagnosed for years and their kids and they’re fine and that’s what I rely on as my positivity. And I think that if someone when they’re first diagnosed gets told about these positive other women, I haven’t found a man yet in this, but positive people then I think it would be great. |
|  |  | Faye | they sent it to the Royal Marsden for a second opinion and the Royal Marsden came back and said it’s Multicystic mesothelioma. […] but they, in the hospital general they didn’t tell me anything, it was just complete and utter silence. I was ringing the consultant’s secretary and she kept saying, ‘No, there’s no news, there’s no news.’ |
|  |  |  | I mean I’ve been told that I maybe have ovarian cancer. […] And then they said, ‘We’ll ring you when we’ve got no, news,’ and no one did and there was no follow up, no one just ringing to say, ‘Are you alright?’ you know, from the hospital. |
|  |  |  | But it was just, yeah, it was just complete and utter … if they’d said, ‘Oh, we’re sending them off to the Royal Marsden because we’re not quite sure,’ at least you think, ‘Okay, they are doing something.’ […] But then to hear nothing it was just, you know, awful. |
|  |  |  | even before they knew it was mesothelioma, I think the way the staff at my local hospital treated me was awful. […] You know, to tell someone they might have ovarian cancer and then say, ‘Oh, we’ll be in touch,’ and then just not contact at all for probably, it was probably about six weeks, I think. […] And they were very long weeks. I think that was really bad, really bad. |
|  |  | Gladys | I had a lot of bouncing around and I had to do a lot of advocating for myself because it wasn’t a smooth process should I say my dealings with the NHS. |
|  |  |  | it was traumatic at the time and it was horrendous at the time because you then start looking at yourself and you’re thinking, ‘Well, am I doing something wrong? Am I saying something wrong?’ And then you think, ‘No, you’re not, don’t doubt yourself, you know how things should be this and this is not how things should be.’ |
|  |  | Clara | why is it so different from what one person saying to another one? And they haven't even got the biopsy results yet. |
|  |  |  | I was just like, why would you say that to somebody? Like, why would you bring them up so much when I would have been positive myself that they hadn't found it in my bowel and my bladder. Like they didn't need to do that. It didn't need to like come and try and blow smoke up my ass and try and make me feel better, when they weren't 100% of what was going on. Like it was really strange. So, you've got like the two completely contrasting. I'd rather they say to me, just wait for the biopsy. Like I'm guessing right now. Like, I'm guessing and that can make you feel worse or better. |
|  |  |  | It was just like either really, really positive or really, really negative. And now it's turned out to be none of what they were saying it was. It's just like, like what I was saying to her like why are they doing this? |
|  |  |  | so like confusing and so different from one person to the next. […] I mean, the nurse, the gynaecology nurse that phoned me when she was saying it's really bad. She's looking at it from a gynaecology perspective. And then when I spoke to Sam, we told her what this other nurse had said. And she said, looking at it, and me being the actual specialist for this type of cancer that we now know what it is. She was like, I don't know how she got any of that. But she'd obviously read it in terms of her, like department. |
|  |  |  | the surgeon came to speak to us. I mean, what he said was wrong anyway. So, it's no, it's not been like, this is concise. This is what's happening. This is like definite. And all I've ever really wanted the whole way through is just like the truth. Like, I want to know what you know, I don't want to know what you're gonna guess, like. |
|  |  |  | There's gonna be more of like, it's not been as simple as just saying to people, that I've got cancer and that's and this is what cancer it is, and this is what's going to happen. And I think people find that really challenging as well to support me when they don't really know what's going on. Does that make sense? |
|  |  |  | So, all this, it's all like had a knock-on effect, isn't it? With like, making it even more exhausting for me and for them, and then probably just being really confused because they might even be thinking, like does she even know what she's talking about anymore? Like, are they even like, has she even got cancer? Is it gonna turn out to be something else? |
|  |  |  | Like, even just the other day, it was like a doctor's appointment at a certain time. And he always gets things wrong. Like, I'll just like put everything in my phone. I know what he's like. And it's he's tried to like, turn it a little bit like he tried to say like, oh no, it's not at that time that you're saying you're just drugged up, like turned out I was right. […] Like, just silly things like that. Like, people just brushing off things that I say now, because it's gone on so long. |
|  |  |  | it's just knowing all of the biopsy results and everything. And I don't, and it starts to creep in that maybe they're not saying much to me now. And keep saying, oh no, wait till the meeting, because it is really bad. It’s just your mind goes from one place to the next. It's like, maybe they're not telling me the truth. Maybe they can't do anything for me. But they just like, need to discuss it. And like get their ticking the box and their monthly meeting before they can actually tell me for sure. So yeah, it's just really like confusing time. |
|  |  |  | I mean, the trouble of the diagnosis for me was that it was like, so like, wrong for so long. […] Rather than just like getting upset about things, and it turns out not to be that anyway. I know, that's quite specific to me. But there might be other people that is, you know, I, I've seen some things where it takes a long time for it to be diagnosed. Because it gets confused with other things like mine was with the, the IBS, and then the ovarian. So yeah, it's kind of it is, it's really difficult to just say, but I wish I hadn't taken everything as like, verbatim. |
|  |  |  | you just think with like doctors and nurses and things, you just like trust what they're saying a lot of the time as well. It's like, they this isn’t my job. I don't know, they know more than me. So yeah. So, you just kind of like put all your trust in them. It's horrible not to put your trust in them. But you probably shouldn't put all of your trust in one person like that. And in that moment, believe every single thing that they're telling you, especially if they haven't got anything to back it up with. |
|  |  |  | So, with this rollercoaster that I said to you about one minute thinking, I've got like leukaemia and then I've got ovarian that spread, that's really bad. And I was still googling things at that point. Which is really bad. |
|  |  |  | the gynaecology team at the other hospital, […] I said, can you just give me a diagnosis and they’d taken one look at it and said, we think that you've got ovarian cancer that’s spread |
|  |  |  | literally had got to the point where I thought like, no one phones you back. No one knows what they're gonna say, what they're gonna do. No one does anything very quickly. |
|  |  |  | went to the worst places. And so, did [L] as well. We're just like, oh my God, what's happening? |
|  |  | Tom | So initially, just after that surgery, they thought I had Pseudomyxoma, so cancer of the appendix. But the taking it out, the worry was that they'd found, like cysts on my peritoneum and some fluid as well, which was unusual. |
|  |  | Mia | I think most women, they go in with pains. […] It’s just, it's awful, especially if you feel like you've got history. They like, they make you feel, it's proper bad. They're like, well, we're going to test you for chlamydia. We're going to test you for all this. I'm like, I’ve been with the same partner for like, you know what I mean? I'm married, I've been with him for like three years, I've definitely wouldn't have anything. They’re like, well, we have to be sure. And then obviously, so they do all them tests make you feel awful. But then obviously, you've got to wait two, three weeks for them to come back. |
|  |  |  | And then it's oh well, they were all clear. So, we're going to send you for bloods and then stool samples and then a scan, and then obviously have to wait like seven weeks my scan. |
|  |  |  | the scanner man said they should have sent you straight for like an MRI or they should have sent you straight to this day one kind of thing. And that made me worry because I’m like, oh what do you mean? But then obviously, then I had to wait for more. I’d literally, I didn't have my MRIs until after my surgery. But if, if I didn't go in there crying, it would have been months later and later, I think. If I didn't obviously, pretty much act like I was going to die, if they didn't do something, there and then there. |
|  |  |  | I feel like I wasn't getting heard. And every time I rang the doctors it's just the same like, oh well, you know, we'll, we'll try and get someone to call you back. |
|  |  |  | But I was just kind of glad to finally get a diagnosis because obviously, I've been told so many different things. And I did get told at one point that it was like cancer, and I had the cancer lady come into me. So obviously, I've already had that stage of feeling the worst, thinking the worst, crying. |
|  |  |  | When I had my first surgery, obviously they all was under the impression, I think being a woman they're always like, right so it's a cyst, you've got a cyst. It's all they always say and because I was in so much pain and I was there, and obviously it wasn't an urgent thing the cyst, you know we'll sort it out eventually. |
|  |  |  | Being a younger person, most of the people on these support groups are obviously a lot older. […] Because a lot of them obviously, like, I think they've got it through like, actually people used to work with asbestos didn't they? Obviously, many years ago and they used to wear like suits and stuff, and it didn’t really work […] So, obviously a lot of them are older and that. But you do feel a bit I don’t know. I guess, I don’t really know how to explain it, really. Like I, I won't really go into these meetings and stuff like that, because obviously they are a lot older, and there are people who will just look at me like. |
|  |  |  | But they have like a meeting like last month and they say, oh you want to come and stuff, at the hospital to find out more information and that. But felt so awkward there. Don't know why, I just was. |
|  |  |  | there's such little research into this one, even though there is little research into the normal one. This one's really even more like |
|  |  | Natalie | So then obviously, I immediately go on Google and start researching stomach cancer. […] And, and then I’m researching bowel cancer, and I just didn’t know ‘cause they phoned me up when I was at work and said that. And then, which is, I felt quite bad. Why didn’t they tell me? Why didn’t they just ask me in for another biopsy if they didn’t know, and not … it’d have been better if they didn’t tell me anything until they knew. ‘Cause all it did were make me, at that point, really worry sick, and nearly made myself poorly worrying sick. […] I, it really made myself sick for a good few weeks while they didn’t tell me anything. I think it’s better to not know than, than know that … |
|  |  |  | … ‘cause the stress is more horrendous, I couldn’t sleep, it was awful. I was having headaches. And it, ‘cause I get migraine headaches if I get stressed, and I didn’t get a migraine at the time, but I got really bad headaches and everything, and yeah. It causes a lot of stress not, the not knowing. |
|  |  |  | we went ski, we went to, we went to Prague in, in January, and then we went skiing, and then, they says, ah, yeah, he’d rang up and says “You’ve got cancer.” He rang me up and says, “You’ve got cancer.” He didn’t tell me anything about what kind of cancer. I thought, “Oh, well, that’s great. What kind of cancer?” |
|  |  |  | then I got another letter saying to have a, an, to go and see him, and then he said, “Oh, I don’t know where the cancer’s coming from. You’ve got cancer floating around in your abdomen. Little cancer cells.” |
|  |  |  | ‘cause that woman when I went in January said you can’t see your ovaries, so I assumed it was some kind of ovary cancer, and that’s why I can’t have kids, kind of, thing, but nobody’s told me why I can’t have kids. […] And then I went to see a different oncologist |
|  |  |  | They took it, sent it to Hull Hospital, and they, it was inconclusive, the sample was. So they said this cancer, it’s cancer, they just said it’s cancer. And I said, “Well, where’s the cancer from?” “I don’t know. It might be your stomach,” |
|  |  | Brian | it really was a case of getting me in, getting me open and finding out what, what it was. They, it was never well, you've got bowel cancer, or you got bladder cancer, or, or it was never like down to a specific type of cancer |
|  |  |  | When I had a tumour cut out of me in the January of last year, it took a few months for them to identify what it was |
|  |  |  | The diagnosis was after the tumour had been removed |
|  |  |  | I received a phone call saying that I'd been diagnosed with this. And then I was asked to go up to the, the Cancer Centre up at Guy's hospital. […] And there I, I sat down with Professor S., who went through it all in the presence of a meso nurse. So, it was, it was sort of like March, April time is when it all started to become clearer as to exactly what the situation was as to what you know what I actually had. |
|  |  |  | so that was removed. And then as I say, a few months, it took them a few months to actually identify what the mess was. |
|  | Subtheme 1.2 **Loss of Femininity and Fertility** | Hannah | You know it’s the stuff that happens afterwards. We can’t have children because I had to have a total hysterectomy. So, I’ve no ovaries, no womb. |
|  |  |  | I think from, like, the hysterectomy as well because we don’t have children and, and now we can’t have children naturally and I think that’s a, sort of, challenge that you get being a younger person, I guess, when you’re in your sixties you, you, you don’t … fertility is not something that, that’s on your mind. |
|  |  |  | And the changes it makes in your body afterwards. I mean, on a sort of, sort of, sexual relationship level it’s not really the same as it was before and, yeah, it’s basically, because your, your body has changed. So, I, I think that’s quite a significant challenge and, and I think in general there’s, there’s a lack of support out there for things like that. |
|  |  | Sofia | I can’t have children and that’s a big thing so. And I’m now menopausal as well, I’m in the menopause and that’s a big thing. |
|  |  |  | when you’re only 30 you think, ‘Oh, I’ve still got like another like …’ in my head I think 100. I’ve still got another 70 years left, that’s a long time to live still. And so like for women like, like to get the surgery you’re fertility so that’s something to deal with. And to face that you might not be able to have children |
|  |  |  | you’re facing mortality and your life is just basically going in a different direction of basically what you get told when you’re growing up. Which I think is quite bad that you get told that you need to go to Uni or go and get a job or an apprenticeship and then you need to be successful and then you need to go and get a house and then you need to go and get a family and have lots of kids and all this sort of stuff. But then I’m just like oh, I’m not following the norm |
|  |  |  | So I struggle, I suppose the, the meso … with the, the menopause thing that was a big thing just because it took a while for like the hormones to kick in |
|  |  |  | not being able to have children, I kind of, is difficult but because you don’t really know, like I was never one for like was I definitely going to have children or I was I not. But it’s like that option’s now gone and it’s difficult |
|  |  |  | when I first got diagnosed I was a bit like embarrassed by getting diagnosed as well it was like a sense of like I was embarrassed by this, like I was embarrassed the fact that my body’s letting me do this. I was embarrassed that like it was kind of like weak a wee bit. I know no one would ever say that but it was like I was in, like I’m embarrassed to like tell people about it a wee bit sometimes that I had cancer. |
|  |  |  | and then like because obviously it’s like when you tell people you have cancer it’s the whole, ‘What did you have?’ And then kind of embarrassed to say that, I don’t know like as a 30 year old like I’m now like menopausal is a bit embarrassing a wee bit. |
|  |  |  | because see when you go through chemo like your face changes, like it blimped up and like I lost some of my hair at the front so I was like looking in the mirror and I was like this doesn’t even look like me. And then so I think that’s the reason why I was like getting into like all vanity, go and get my hair done, go and do all this. And then now this is why I get all these facials which I never done before because I’m like this paranoid that I’m going to wake up one day and I’m going to look about 90 years old, you know. Because like your skin loses all the collagen and I’m not, could just know that those hormones that I used to have. So I’m always a bit wary of that so I’m a bit embarrassed because it’s like if I mention like what I went through people will know that I’m menopausal and it’s just a bit … But then I’m like, so I think that I’m just not there yet |
|  |  |  | I’m thinking of getting like a tattoo so kind of like so I don’t look at it, I’m not reminded of it. So I was thinking of getting a tattoo to kind of just cover it up so, because I’m quite conscious of it a wee bit sometimes like so when I’ve got yoga I’m always wearing big baggy t-shirts which I probably don’t, wouldn’t ever used to. But I’m now, I’m slowly getting there so sometimes I think I need to embrace it, I just don’t want the questions. |
|  |  | Faye | Well, from being fit and active, you know, from … And also, a complete and utter total hysterectomy when I was, you know, I had, I wasn’t even in the perimenopause stage […] that was really difficult to deal with, it is really difficult to deal with. I’m on HRT for it. […] I think it’s a lot harder if you look fit and healthy. |
|  |  |  | that’s really hard. I mean I think for, obviously there was no way I was going to have any more children but for a woman anyway you’ve got to get used to the fact that you just lost all your childbearing ability. […] And does that make you …? I don’t know it’s less of a woman. It’s quite hard and everyone’s quite kind of matter of fact about it […] and you’re thinking actually it’s, it’s changed me I’ve, you know, I didn’t want any more children, but I couldn’t have them if I wanted them. |
|  |  |  | I think particularly for younger people as well they’ll look absolutely fine […] and that can be quite hard for them to deal with, so like with me |
|  |  | Gladys | The nurse that, the nurse in the hospital, Mr. [U]’s nurse is called a colorectal nurse. I said to her when I was on the regular ward, ‘Now, you’ve removed my ovaries should I now be speaking to somebody about HRT?’ And I was dismissed |
|  |  |  | I cannot believe the things that I have been worrying about, about the way in which my body has been ageing. Which, you know, the little person in the back of your mind is saying, ‘It’s cancer, it’s cancer, it’s cancer, it’s come back.’ Wasn’t … It’s because like I said to that nurse, ‘Should I be speaking to somebody now about HRT?’ And she boohooed me. |
|  |  |  | I think is a failing, is they look at you and they see, the NHS now they see the cancer therein and they’re great at getting the cancer out. They don’t look at you … I think the word is holistically, is that right? […] There’s not a holistic approach, they merely see you as the tumor. And let’s get the tumor out and off you go. |
|  |  |  | I just think that they need to get their act together and look more holistically at it, you know, we, the, the cancer. It’s great they’re prepared to take our cancer out or to treat our cancer to reduce our cancer but the surgery or the treatment can actually cause other issues that can have the longstanding effects. |
|  |  |  | I’m constantly reading about women saying, ‘Oh, my joints, my joints, my joints,’ and I feel like saying, you know, ‘Have you had your ovaries taken away? Because it might be it’s not the cancer, it’s not the chemo, it’s the fact that your body is starved of what it needs.’ |
|  |  | Clara | so I've, we, on our top floor, we've just got like the bedroom and the bathroom. So, it seems like that's where I spend a lot of my time at the moment especially since I come out of hospital is literally like between these two rooms. But yeah, that's just like you know, I, I enjoy getting up and having a shower after the night sweats, having those baths and then there's the other side of it was like the bump type thing when you go in there and then there's a long mirror, and it's just like there's no getting away from it sometimes, like if you're getting ready to have a bath or something, it's just like. Like, I look massive, I must have like loads of fluid and things like that. These things that go round your mind. |
|  |  |  | I think I took it initially because like frustrated that day. Like trying to look and just be like, oh my stomach looks massive. It just kind of go, go in and out of that. Like, oh it's not that bad one day. And just try not to concentrate on it too much. But then there's I think the day that I took that, it was like annoying me. Just like, and there's nothing I can do. |
|  |  | Mia | I feel a lot of people my age, they are obviously going to be more effective by the body image thing. Luckily, I am married, and I've had three kids so I'm not too. What have you lost? I'm not too bothered about my looks but obviously after this, even I won't be wanting to wear a bikini. |
|  |  |  | So, for a lot of younger people that maybe won't want to model as a career or really focused on their looks a lot to be an influencer or anything like that, which nowadays I wouldn’t do. Then obviously it’s gonna affect their, it’s gonna knock their confidence massively. |
|  |  |  | I feel like yeah, it's gonna knock a lot of their confidence |
|  |  |  | Then me just before and thinking, oh God, I might lose me ovaries now might never be able to have another child. I've already got three boys. But I've always wanted that little girl do you know what I mean? And I'm young still. |
|  |  |  | I've had my kids at 18. Well, my first. But I’m kind of glad I did now because obviously, I'm not going to, if I didn't have any kids already, I'm 26. I’m gonna lose everything the next week. So, I wouldn't have been able to have a family. So, I guess, for other people that decided not to and then they like, if they was in my shoes, and they hadn't had any kids it’d probably be even worse for them because it’s worse for them because they know they're never going to have family. |
| Theme 2 **Psychological Distress** | Subtheme 2.1 **Ongoing and Invasive Anxiety** | Hannah | very negative actually. There isn’t much good news out there about mesothelioma |
|  |  |  | It was anxiety with a small amount of post-traumatic stress that I would say. Like I would have like flashbacks to like the surgery, have nightmares that I’d have to go through it again. So, there was an element of like post-traumatic stress, but the majority was anxiety. Feeling overwhelmed, that I just couldn’t cope with things, and very emotional. Never happened before. |
|  |  |  | So, I feel that I live in the worry that it’s going to come back. So, they told me before the surgery that even after the surgery there’d be a 50% chance that it would come back. A 50% chance is pretty high and my worries from that is if I’m in the 50% where it comes back do I have to do that surgery again, because it was the worst experience of my life. And if it comes back and they say, “We won’t do the surgery again,” then do I have to rely on chemotherapy and immunotherapy, because from what I’ve seen it only lasts for so long. And then it comes back again which, which is not great. So, I think … And, and while I want to be positive and I am generally a positive person and I, I put myself in the 50% where it’s not going to come back, I do of people that have had the surgery two or three times and that is a bit of a worry. |
|  |  |  | I was told that we never really go into remission, because, yeah, they can remove the cancerous cells but not the asbestos fibres, they’re going to stay there forever. So, yeah, there is a constant worry about that, because you, you know it’s possible it’s going to come back. |
|  |  |  | I feel that I don’t want to hide away and be protected forever. But I’m also aware that I’ve got this 50% chance of it coming back looming over my head. |
|  |  |  | I think it didn’t help that being off sick you don’t really have a lot to occupy yourself with, right, so you tend to think a lot more. |
|  |  |  | I think the problem was that when you don’t have any sort of structure to your day, you get bored. Then you are thinking of, I don’t know, maybe you do Google searches for things you shouldn’t be looking at, or if you are basically on social media all the time it’s not really good for your mental health. |
|  |  |  | Like I actually don’t know how people do it, the ones that don’t work and are at home all day, because I honestly, I was going crazy. Like I hated it, hated being off sick. I must be the only person that ever says that, but I hated it. Yes. Just, just not so keen on my own company. I like being with other people and, yes, it was most definitely not for me, and I couldn’t wait to get back to work. |
|  |  |  | when I did go back to work, work was like my escape from the anxiety really. It was the time where I wasn’t thinking about things like that because I had to focus on my job. So actually, I think the best thing I did was go back to work. It certainly helped anyway. Work were brilliant. We did like a slow return to work. So over like a 12-week period I slowly increased my hours. They were fantastic really. |
|  |  |  | Definitely like the mental health side of things, because it distracted me. You know if you are focused on counting how many stitches you’ve done, then you are not thinking about other things. |
|  |  |  | Distraction techniques are the best, I would say. Much easier than actually dealing with the thoughts |
|  |  |  | I think it did really help me. Yes, I wonder how it would have been if I, if I hadn’t been able to distract myself with yoga, craft work and things like that. Yes. And even now, like there’s some weekends that I still really do struggle if I don’t have anything like planned to do. So, I am like currently trying to fill my weekends, because I know that’s when I struggle the most |
|  |  | Evelyn | it was a bit of a shock diagnosis |
|  |  |  | I think just for those first few days just not, not quite knowing what to do, a bit of a sense of … I didn’t let myself dwell too much on what could be the possibilities, I think. It was obviously the initial shock and I, and I, I think it was probably one of the few periods where I was tearful |
|  |  |  | I think it was shock and it was very strange, a very strange feeling because I just wasn’t used to that, that sense of not knowing. I’m normally quite well prepared for most things in life I think and that was a shock. |
|  |  |  | when it happens to you. You’re so used to seeing images on the TV or reading about it you think you would be prepared for a situation but then it actually is, it’s very strange and it doesn’t feel real |
|  |  |  | I think you’re prepared for most things in life, you kind of have experiences that lead you to things and this doesn’t, this didn’t, there’s nothing quite prepared me for, for that, |
|  |  |  | It was very, very, we talk about, we talked about a surreal experience of the diagnosis but that seemed very surreal as well. […] The treatment, yeah, well the operation. The time I was, I was down there Mike came and stayed in, they provided accommodation for him down in Basingstoke as well. And I think for him it was a strange time as well |
|  |  |  | mentally I’m aware that it’s there, I have, you know, I’ve had the treatment, at the moment there’s no evidence of disease but there’s that consciousness in the background that it’s, it probably will reoccur at some point. And so that’s, that’s sort of where I am at the moment with it. It’s just there all the time really |
|  |  |  | like I say, it’s always there, always thinking about it. |
|  |  |  | I’m not overly anxious. Like I say it’s just those thoughts pop up |
|  |  |  | It’s just particularly thinking about things that are going to happen in the future […] and not knowing what, what’s ahead. So when we’re planning ahead for, for what, for holidays or what we might do with the, the kids and things like graduations and things it’s, it’s, there’s that question, it pops up as if I’m going to be there or what kind of, what will my health be like at that point. |
|  |  |  | Unfortunately, she [sister], she died last, at the end of October so […] that’s been quite hard really too because she was there just for little messages and to talk. And, and seeing her experience and obviously that, that’s brought up a lot recently for me a lot of worries really about what’s ahead. |
|  |  |  | But obviously I feel like things are, are cut short a bit so it’s difficult to kind of make plans for the future. So there’s that impact. And I, I think I do worry more about if I’m ill. |
|  |  |  | it’s just that uncertainty as to what’s ahead. I dwell quite a lot on, am I going to see the next stages of the kids growing up in particular? So am I going to know what jobs they will be doing? Their future partners, their children, just sort of, they are all things that I think as a parent I want to know, you know. But I think there’s an urgency to sort of see it, you know, and to see it through to, sort of, you want to be there for those key moments. |
|  |  |  | And I think that’s been emphasized quite a lot recently by, by my sister Tasha’s death that she, she’s, her children are the same age as mine. […] And they are, you know, seeing what they’re going through, and will that be the situation that my children will find. And, yeah, it’s that sort of wanting to know what’s ahead for them a bit. |
|  |  |  | one of the photos was of that trainline it was, it’s what’s down the track, it was going to be a sort of, I was trying to sum up whether to do a picture of the kids going with a rucksack on their back going back to Uni. It’s that, yeah. And it’s not knowing what’s ahead really for, for me and them really. |
|  |  |  | But it’s just that problem, again looking forward, what if I, if the, the disease reoccurs is it going to prevent me from doing those things I enjoy which is eating, walking, well, and you know, if I can’t get out as much |
|  |  |  | I think thinking about the future and, and how, and I mean, essentially think that it might be involved starving to death really if, if it does, the impact of peritoneal being that, that you just can’t get enough nutrition in that, that does frighten me a bit. You know, and I think that, that comes from, from, I don’t suppose people think about how actually how cancer kills you often and what, you know, what it is that actually kills you. And was highlighted again by my sister I think realizing that with her case it was liver disease, it was the… and the liver failure caused as a secondary cancer |
|  |  |  | So realizing that it’s not what you say, like with her case with it being bowel cancer it wasn’t that that killed her in the end. And I think it’s sort of like then thinking about how, yeah, how you die and how unpleasant it is… so the idea, yeah, of, of something that I really love, you know, food and eating is going to be something that I just won’t be able to do and then in the end |
|  |  |  | But that’s sort of really what that represents is sort of not knowing what’s further down the line and not knowing what’s ahead with the, you know, with everyone else. |
|  |  |  | The food one is, is very much it’s sort of like how much, you know, I enjoy it and it’s, I mean there it was a social, you know, it’s a social experience. And it’s … But how, you know, how the disease has impacted me and might in the future with, with regard to food, so. |
|  |  |  | it pops up all the time really […] and then often at night, you know, if I wake up when your brain starts going through things […] and I think about, a lot about it then, yeah. |
|  |  |  | the sleep one is, is about in waking up in the night and the, the, the worst thoughts pop up. I have had a lot of sleep issues really and I regret (?) that’s probably, you know, the great, one of the greatest impacts. […] I’ve really struggled, obviously the first diagnosis to sleep, there were long periods when I’d be awake |
|  |  |  | I was in the hospital for 11 days which is relatively short apparently (laughs), but it still seemed a long time. And the whole time I was there I didn’t, you know, didn’t really sleep properly. And since then, probably one night a week when I get a good, good night through […] some nights it’s not so bad it’s sort of … Because I’ll wake up every night but some nights it’s easier to go back to sleep than others |
|  |  |  | I think, well a lot of it is distraction. […] Yeah, probably just a sort of like, ‘Right, we’ll park that for a bit and get on with …’ Because it [anxiety] pops up, yeah, it inevitably pops up at times |
|  |  |  | So yeah, it is that sort of, they are a source of support themselves but, yeah, a good distraction as well. |
|  |  | Sofia | I got told that all I only had one to two years to live and rightly or wrongly whether she should have told me that is a different question |
|  |  |  | And then I met my oncologist and she said, and he did say, he said, ‘Oh, she’ll go in, you’ll go in there and you’ll meet her, and she probably will give you like numbers and things.’ And she said that I had one to two years left and I just, I do still struggle with that, and I have that conversation with my therapist a lot. I have mentioned that before as to like a way not to have that conversation with people, I think they should, I just don’t find that useful to anyone being told that. |
|  |  |  | I suppose like anyone I wanted to go and run, run out in front of a bus when she told me that. |
|  |  |  | there’s no, no harm like sitting in front of the TV but sometimes like when you’re sitting where you’re still you tend to think so it’s hard to get away from it. |
|  |  |  | I mean watching TV because sometimes the TV triggers a lot of bad things like it’s amazing like how many adverts are about cancer or about illness or you watch the news or so I don’t watch adverts anymore. I don’t like certain TV programs, you start off like a superhero film and all of a sudden someone’s been diagnosed with cancer and you’re like … And before it probably would just like, I’d probably just like not even think, but now I’m like … so I kind of like tend to avoid kind of programs like that. Like that program Afterlife I would, I probably would have watched that before but now it’s like oh it’s probably the worst program I could ever watch because it’s probably like me |
|  |  |  | It always, the same memory that always goes, I go back to is that one-to-two-year thing. So I, I rarely think about my surgery and the chemo and I’ve kind of like, I see them as like getting to this point. But I always go back to that one-to-two-year conversation |
|  |  |  | But, and I always go back to that, that one to two year thing because when I have a bad thought I always go I haven’t actually reached that two year spot yet technically it’s supposed to be in October since she told me that conversation that’s what I’ve relayed it to |
|  |  |  | my therapist thinks that it’s an element of PTSD that I go back to that because like when I reached, I had to go to the hospital, every time I had to go for a scan just a routine scan and like as soon as I walk in the place I could just, it’s like it’s like someone’s sitting on my chest and I just like can’t talk and if someone even talks to me I just can’t like |
|  |  |  | I struggle going into Victoria just because it reminds me of when I got chemo and all those conversations. And like I’ve had like multiple scan, I’ve had so many scans and I just, I just, I just feel as soon as I walk in the place like it’s just not a good place for me and it just feels … And as soon as I go out I feel like, ‘Oh, I can breathe again.’ Like it just, I just go back to that just remembering the whole, the whole like conversations where it goes through my mind as soon as I enter that place and it’s very difficult to get it, to not think about it because you’re actually in the place where she told you that conversation, so. |
|  |  |  | And that building’s, like I could walk to it in like five minutes I could, I just don’t live that far away from it. So however, it’s one reason why we’re moving a wee bit further out so I’m not like walking by it all the time. |
|  |  |  | one thing that we’ve worked on at therapy is the, like what triggers because I never really thought about it like what was triggering and she said I have to think about it. So ever since she told me to think about it, I’ve been thinking about it as like adverts, like social media as well it’s like Facebook seems to know that I had cancer for some reason. And it’s not like I actively like seek out all these places, it’s stuff like I get all these adverts so I like decline them |
|  |  |  | Conversations face to face doesn’t bother me actually, like that doesn’t bother me talking about it. It’s probably just like when I’m in quiet time […] is when I try to avoid, avoid it. |
|  |  |  | Now I’m back at work like through the day I’m always just like thinking about my projects which takes my mind off things. And I usually have things I’ve planned the week, in the evenings and the weekends so I just like just try to keep myself busy. And like when I’m with Mark my mind doesn’t really like wander and if we’re watching stuff, it’s fine. So, yeah, I have got a lot better of like trying to like not think about it |
|  |  |  | we’ve been doing exercises about thinking about the future and trying not to go back to that memory. |
|  |  |  | So I think that’s why like one thing can come from this is that initial conversation like I’ve got over most things but that initial conversation is still lasting with me… all these years. |
|  |  |  | It’s a rare cancer so it’s difficult for everyone so I just think as long as there’s support at the beginning not going told that bad element of what the oncologist told me I think |
|  |  | Faye | I don’t like talking about it, you know, to other people, obviously taking part in an interview like this is fine. I’ve tried the online group but I don’t like it because it’s triggering for me, I, I try to compartmentalize it and put it to one side. It’s just very hard, I mean I was treated for PTSD after I had my operation. |
|  |  |  | because in some ways I have to explain what it is again and again and again. I find it quite triggering, I mean I’m a lot better now because I’ve been treated for PTSD. |
|  |  |  | The PTSD really was manifested, I mean I had already been referred, but it was really bad at the start of the pandemic because there was a lot of imagery of intensive care units […] with medics all in full, you know, dress in, in their scrubs. And that was very distressing for me because it brought back horrible memories just, you know, I couldn’t watch the news. You know, just talking about it now has given me a slight flutter so, so it was that kind of, that’s why I didn’t like to think about it, it’s much easier to compartmentalize it. |
|  |  |  | And bringing back memories, I mean particularly for me it was the, the tube down the throat which I just, you know, I can imagine it and then physically start gagging it’s really hard. […] And just the, you know, memories of bleeps, machinery, that sort of thing. […] It’s very hard. |
|  |  |  | a friend that I used to work with at [Broadcaster Z] was diagnosed with, I can never remember the name, pseudomyxoma, the, the appendix cancer. […] and at the time that he was having his major surgery, exactly the same surgery, exactly the same treatment plan it was very triggering for me because that’s when I was actually having my PTSD. |
|  |  |  | I mean it’s really hard, it’s really hard living with this |
|  |  |  | I mean it’s upsetting, I’m in a, I’ve always been very physically fit and active which stems from when I was young, and I had kidney problems, so I find it really upsetting that now I have to take tablets. […] now I have to rest, now I have to some days just say, ‘Oh, I can’t do anything,’ say, ‘I’m absolutely shattered I’m going to have to sit on the sofa for the afternoon.’ And I, I really don’t like that, I really don’t like that because I, I don’t want it to be me […] and it is me. |
|  |  |  | there are still loads of sleepless nights when I’m Googling it at three in the morning |
|  |  |  | My life’s changed completely. […] it’s a lot quieter now, my world’s kind of shrunk. I don’t do as much mainly because I’m so tired, you know. I don’t socialize as much, yeah, I’d say different […] I lost confidence in my body |
|  |  |  | because I felt it was absolutely fine and I was, you know, running the Great North Run or whatever, but all the time I had this thing growing inside me. So now, you know, if you get a niggle and you worry about your niggle. Yeah, it’s changed me a lot I’d say. |
|  |  |  | I suppose it is a mental process as well because quite often I’m having a little mental battle within myself about how much headspace it takes up when you don’t want it to. So you have to kind of box it up and put it to one side and then just get on with everything. |
|  |  |  | when I had my online appointment with Mr [M] in the March he said, ‘Actually it’s showing a two-centimeter cyst on your, on your liver.’ So that was a complete and utter mind fuck |
|  |  |  | I had the, the scan in May and it showed up three spots so liver, colon and just, what’s the other one? Bladder, that’s it. So that was really really hard to deal with. And going from no evidence of disease for three years effectively to 21 to have it back three times that was really hard. […] I do have this little niggle about what are they going to find in October? |
|  |  |  | you know, I’d be sitting in meetings and drifting off or trying to write emails and staring out the window and just trying to, just thinking about it the whole time. […] I don’t want another operation, you know, my God, what I went through, but I would obviously have to have it. |
|  |  |  | I’ve got nagging at the back of my head, well, yes but it doesn’t respond well to chemotherapy and, and what happens if it’s not slow growing? And what happens if in October they find it’s everywhere again? |
|  |  |  | my world has completely shrunk. […] So, you know, from, it’s just gone like this to it’s just now me and my husband, both my sons have left home and I don’t put myself out there as much. So, you know, if I was stopping work before mesothelioma I would have been thinking, ‘Right, I can volunteer for that, I can do this, I can do that,’ and now I’m thinking I don’t know, I’ll just wait and see. |
|  |  |  | The support there coming up to the scan, you know, is, is there on the horizon now even though it’s going to be October. That’s, the closer it gets the more difficult it gets. |
|  |  |  | the hardest thing for me actually after the operation when you’re waiting for the first-year scan because they don’t do it until a whole year has gone by if you don’t know how successful the operation’s been. |
|  |  |  | But then when you actually have the scan and they tell you it’s NED no evidence of disease and then you’re thinking well, you want to be cured but you’re not cured because you have to continue to have these monitoring scans. So, you know, that, that’s quite hard, but it’s always there. |
|  |  | Gladys | I cannot believe the things that I have been worrying about, about the way in which my body has been ageing. Which, you know, the little person in the back of your mind is saying, ‘It’s cancer, it’s cancer, it’s cancer, it’s come back.’ |
|  |  |  | It’s like in my DNA and altered my DNA and it’s like something that could come back.’  […] so sometimes that is in my mind |
|  |  |  | I can play devils advocate with myself and say, ‘Well, you were never ill before you, before you realized you had mesothelioma,’ so a lot could be going on |
|  |  |  | I have found it mentally more traumatic. The physical aspects have been easier to deal with than the mental trauma … |
|  |  |  | huge anxiety, huge anxiety, yes, massive, yeah. Because of course, what it leaves you with that condition I’ve got |
|  |  |  | because of the scan and even, you know, and we all get down sometimes, don’t we? And you, I know and you, you can’t help but sometimes … I think anybody can sometimes have that |
|  |  |  | what I try to explain to someone is, is like if I had, if I had had a severe leg break when I was in the police that caused me to have to retire early and I had so many pins put in my leg I would probably walk round with a limp and every now and again that limp would get bad depending on what life threw at me. Yeah? And that’s the same. I had a severe case of anxiety where I couldn’t function and every now and again something life throws at me will bring it back and, and, yeah, it is like, it’s not nice, I don’t like getting it. So I, I, when I … I’m exhausted by it. It exhausts me because it just consumes my whole being. And it’s a horrible thing because there’s me here, I’m still really nice and then there’s this thing that like vile. And that’s the anxiety. |
|  |  |  | I had my scan in November and I think it was March by the time I’m finally told, ‘Oh, yeah, everything’s okay.’ […] (laughs) And that … yeah. So I’ve had, I’ve had a conversation with Mr. [U] and I’ve said, ‘I don’t want one done every six months,’ it’s a slow growing cancer, I can’t cope, I want my life to be normal. I don’t want to keep being building up to having a scan and then waiting for the result |
|  |  |  | like on these Facebook sites, particularly the American one, I mean they’re so fortunate. I know they’ve got to pay for it and we’re lucky we don’t have to pay for it directly, it goes through our taxes. But my God they can rock up for a scan in the morning and they’re back, then they go and have their lunch in the hospital and before they’ve left the hospital, they’re being told the results. […] You know when they think have scanxiety they want to be an NHS patient. |
|  |  |  | the wait, the wait after. […] the wait after, yeah, yeah. It’s horrible, it’s, it’s cruel. It’s cruel that it’s, it’s permitted to, to be this long. And I can’t for the life of me understand why it takes so long for results to come through. Cruel. |
|  |  | Clara | It’s turned into like a big rollercoaster. And now every day seems like, you're on a little one. The whole overall experience has been like, oh, I don't know what's happening. One thing’s changing, and then there's another thing and like the painkillers are working, and I can do this, and I can do that. To, it's gone on so much now and I've been waiting and the pain relief’s, you know, not working as well and things. |
|  |  |  | it's turned into, like every single day, it's just like, I don't know how I'm going to feel. And, or what I'm going to be able to do, which is really weird. Like it's happened quite quickly. It's just hopefully other people aren't going to get to that point. They'll be able to, you know, get diagnosed and start treatment and things like that. |
|  |  |  | like every single day. It's probably not even every day, like every hour every day. Sounds like madness. Like if you'd have told me this before like last year would have been like, what? Like this is crazy. But yeah, just one minute you can eat the next minute you can't eat. Like, one minute you can sleep, the next minute you can’t. One minute you feel alright, the next minute you feel awful. It’s just like such a like, up and down. |
|  |  |  | like it represents like a bit of a long road for me as well. It's just like a, just a continuation of nothing at the moment. But yeah, I don't know, I kind of like want to take things as a positive, but I think some of them there are like underlining negatives, if that makes sense? Like subconsciously taking a picture. And it's, it looks like it's just me and like a long road of nothing. |
|  |  | Tom | there was the unknown that we didn't really know that had mesothelioma. We're still waiting to hear from Basingstoke, we're still waiting to kind of go back to them and see, you know, what it was going to be. But also like the long term of, you know, was I going to be well? Was I going to need more surgery? You know, what was going to happen? |
|  |  |  | it's definitely had its ups and downs especially, you know, around that time of my first surgery. And then probably 18 months after that, it's just the, you know, not knowing what the diagnosis is going to be and what the next step is going to be |
|  |  |  | I suppose I didn't really get much relief because I didn't know much about it. And I suppose it's relief in that I didn't need any more treatment, but it’s the now you've got to watch and wait, sort of scenario. |
|  |  |  | I was worried that I had cancer and that, you know, what was going to happen, what treatment would I need, and I was only young and all that. |
|  |  |  | massively anxious about what was coming, what could happen. Stress around it all as well. And it was quite hard around waiting to hear from Basingstoke for the first time because it was just the unknown. So, you know, you’d always be thinking worst case scenario when you didn’t know any different. And no one really had any answers. It was just like a waiting game. |
|  |  |  | I definitely you know, think about a lot of and but you know I have times where, you know, like sometimes you can have like a bit of an upset stomach or you can have like a bit of trapped wind or something and you get like a pain or you know, something that feels like it did and then you just have that worry. I think you know; I've got some pain does that mean you know discomfort? Does that mean it's changing on my back or? So, when, when I kind of have those it's in my mind as well. So yeah, it’s on your mind all the time. |
|  |  |  | I feel like we’ve done a lot in a small amount of time. Most people, the experiences that we've had generally, like over a lifetime, I feel like all of that is kind of happened in like my first 30 years. So yeah, so now we're kind of looking forward to the next journey, I suppose. But yeah, it's just always there, always on your mind |
|  |  |  | I struggled a lot with my mental health. […] Obviously, because of, of the diagnosis and then what happened with the exam. |
|  |  |  | That was kind of one of the things but then just like the worry about my health as well, always played on my mind, like you know, am I gonna live to be 30? Am I gonna have kids? You know, there's so much I want to do, that I might not get a chance to do if it's the worst-case scenario. |
|  |  |  | you always have a tendency to focus on worst case scenarios. So, you know I always remember after my first surgery, saying to mum, like, oh mum, I always wanted kids and you know it’s, am I gonna you know, have the opportunity to now? And that was kind of always playing on my mind as well. |
|  |  |  | I did a lot of bottling up especially right at the beginning, like all my worries and, and things and not wanting to discuss it in detail. |
|  |  |  | not putting that extra pressure on family because you know, they're already worried about you, and you don't want them to worry about your more. But then just for like myself, you know to not, to not like get upset all the time. Because people would be able to see it, you know because it, sometimes I thought it's better to just put it to the back of my mind and not sure why I’m really upset about it. Or it's really played on my mind, so yeah. |
|  |  |  | So, that's why I was a bit reluctant to. But I feel like people would be because they don't want to talk about those things that are playing on their mind. So, you know, if like one of the things for me was, I was worried that I wasn't going to be able to have a family or, if I did, I wasn't going to be around that long. To talk to my family and friends about that, it's quite difficult. |
|  |  |  | So, I think people would be reluctant to sort of be able to talk freely about what's ever is on their mind. But what I would have done before that is just bottle it up and just worry that I wasn't going to be around long enough to do all the things I wanted to do and that play on my mind. |
|  |  |  | just having scans all the time. But you know, that has its impact because every sort of December time, December, January, you know me, me and Holly and the family are always thinking, oh my God the next scam is coming up, and you know hope everything's gonna be okay. And you know, I hope the outcome is, is the same. So yeah, we always get a lot of anxiety around Christmas, January, about that next scan. |
|  |  |  | it's just always in the back of your mind at that time of year |
|  |  |  | And it itself it sounds fantastic. You know, they don't need to do anything more. It's not changing. It's not growing. Just wait and see. But then, you know like, with the scans I'm anxious every year that one of the scans they're gonna say, oh my God, this has changed. We're gonna have to do this now. You know, it's not one of those where the kind of resolves are, they can just take it away and then that's it. |
|  |  |  | there was some sort of relief in that they don't have to do anything more because when you Google it, they talk about, you know, the sugarbaker surgery and all that sorts of things. So not having to do that anymore. Surgery was good. But you know, waiting and seeing is also hard as well. |
|  |  |  | around Christmas, I know that I'm always thinking about it. So, is Holly. And it sort of like, feels like it has an impact because we're, we're thinking, you know, what if things change on the next scan? |
|  |  | Mia | So, then I was Googling mesothelioma. And I think the malignant one obviously, it is awful. So, I, I went into this condition and absolutely the, the meeting with the consultant at Christie's that give me my diagnosis, absolutely bricking it. And then he did say, oh it, it shows mesothelioma. And I pretty much started crying with my mum. |
|  |  |  | Emotional challenges, it’s quite a hard one. But I feel like you got to always think, that it’s you that has to deal with it. It’s, it’s, everyone else can support you and stuff like that but at the end of the day it’s, you're in your own body, you're in your head, it's, it's not their issue really. So, it’s always worrying thinking about I gotta do this. |
|  |  |  | It's all the uncertainties that come with it. That’s like the worry and stuff obviously with, with obviously already having children and stuff like that. And it's obviously thinking about their future. |
|  |  |  | it's like the uncertainty of obviously not knowing exactly of the outcome. And if I will fully recover all that type of stuff. And then still not knowing whether or not it is malignant. That's an obviously another worry. |
|  |  |  | But then obviously, I know that there's no cure for this, this condition disease, and I know it can regrow. And they told me that. They said, obviously, it could take a year, it could take 10 years. So obviously, you just have to have constant monitoring and scans. |
|  |  |  | I used to obviously like smoke, not a lot but a bit. You know, I, with having three kids and that, that was my kind of stress relief. […] And I do enjoy like the kind of energy drink of monster every day. Only one well, not every day, but every other day I’d say. Whereas now I’ve got that much worry about possibly having a heart attack after this operation. Or I'm, literally I can't do anything now. I'm literally like, I don't even know. Like I don't even want to actually have a drink tonight, I’m going for a meal, I might think oh shall I have a glass of wine? I'm thinking well my livers gonna be removed soon. So, probably not. |
|  |  |  | you also overthink, like every little pain, you’re like, oh, you know what's that? |
|  |  | Natalie | So then when in two more weeks came, and she said it’s mesothelioma, oh, I couldn’t take it in, I didn’t take it in, and they’re all saying, “I’m sorry, I’m sorry.” And these other nurses, and I thought, “Oh, well, it can’t be true, she said it wasn’t two weeks ago, why is it that? I hadn’t been anyway near asbestos ever.” |
|  |  |  | it made me a bit mad when I first got diagnosed because I’ve always eaten fruit and veg. That’s what my snack is when I take to work, it’s a banana or apple, or oranges. I always have, like, about four pieces of fruit a day, and it, and, and eat veg all the time, and I’ve never been a smoker. I don’t, I drink a little bit, but I don’t, you know, in a celebration if somebody’s going for the pub for a couple, but I’ve never massively drank, and I’ve never been obese […] and all those things cause cancer. And I’ve still got cancer, and I’ve done my best to not have, get cancer. It’s mean. |
|  |  |  | Well, I didn’t, I didn’t respond. I didn’t, I just went quiet ‘cause I were, it was hard, it’s hard to take. Well, this is happening to somebody else kind of thing, I’m watching it on a drama on the tele. Yeah, this isn’t me, this can’t be me, yeah. |
|  |  |  | I weren’t really paying any attention or listening because I didn’t really believe, believe it. But I, I still don’t actually know what … ‘caus when we went to see the oncologist just before I started my chemo, he said, “Shall we talk about stages?” So I actually don’t know what stage it is, but, because I didn’t wanna know in case … ‘cause if they tell you a certain stage, and then you know that people die of that in, in, in not, in not long time, you, you live to that. I’m the type of person, I can manifest an illness by looking on Google, so I don’t wanna, I don’t wanna know what stage it is |
|  |  |  | It took a few days, and then I just, I was sat on the settee, and I just couldn’t stop crying. |
|  |  |  | And D didn’t know what to do. So then I said, well, I didn’t wanna die because that woman, one of the nurses says, “Ah you, you need to hurry up and get married,” kind of, thing. The one that we saw in, in York initially. And then I, I told the other woman she’d said that, and she says, “No, you’ve got six months.” “So, six months? Well, what, so that means I’m gonna die in six months?” So six months means I’m gonna die in October basically, but I’ve, I don’t know what she means by that. I don’t know if she means I’ll be well enough to get married in the six months or what. |
|  |  |  | Well, when someone’s first diagnosed, it’s awful, you can’t sleep or anything, your whole world’s turned upside down. |
|  |  |  | the worry is, because it’s on my small bowel, which you can’t, nobody can live without that small bowel. So that’s the worry that I just, praying that the, it’s shrunk enough off that bowel that … ‘cause they can operate on the other bit, but they can’t operate on the bowel. So that’s why they did the chemotherapy on me to try and get rid of it off that bowel, ‘cause you, as soon as it takes over that, you can’t eat food, I suppose. But yeah, living with it, yeah, it’s, it’s horrible when you think about it more |
|  |  |  | so the pain from that worried me. I’m thinking it, if that pain I can feel, is that the cancer growing? So you imagine all sorts, but I’m still none the wiser. |
|  |  |  | I was seeing an osteopath anyway. And I was still seeing him for my shoulder, frozen shoulder, but he’s now dealing with my cancer in my bowels. […] he felt around them and said it’s, it’s likely to be scar tissue, still, still repairing, that’s what he can feel. So I hope it’s not the cancer growing, you know, you, you do worry, |
|  |  |  | But yeah, it came then, and it, we were both quite depressed that, well, we got really down about it that they’ve sent that, it’s makes it real … |
|  |  |  | Just knowing that I’ve got it, every, ‘cause sometimes you forget. And, and I just, I’m carrying on, and I’m doing something, and, like when I got to netball, I’ve forgotten. And then when it hits you, it hits you again sometimes, and, like, I’m, having very vivid dreams all since I’ve been diagnosed, it’s weird. Vivid dreams, weird dreams, and then I wake up and when you realise, you forget initially. Just it’s a normal day, and then you remember and think, oh, no, it hits you like a ton of bricks. I think why have I got that wrong with me? I can’t have. |
|  |  |  | it’s always women affected by our time of month, and sometimes you, at certain periods I, I can get really down, but I do, when I look up when I am in the cycle I know that that’s the, that’s the, it is the cycle doing it, it’s my hormones. So I disregard those feelings, but I do get quite down on, on certain times. |
|  |  |  | I felt down a couple of weeks ago, I thought I, I ain’t got any joy in my life, all of a sudden I went really down and thought there’s not, I ain’t got a reason to live, kind of, thing. […] But that passed, and now I thought I’m gonna ring somebody and find, have some counselling, but it passed. |
|  |  |  | I’ve put myself more at risk with the radiation, with the CT scans, with the chemotherapy. I’ve now, if your naturally, like, if you haven’t had any CT scans, you pick up radiation from the, from the atmosphere. So you have, so, so if you have two years’ worth of radiation in two years, I’ve had two years’ worth of radiation in one scan. And I’ve already had one scan this year, and then I’m having another scan. And that’s all that radiation, and it, and it causes secondary cancers. I just don’t want, you just don’t want … is it really necessary to have these scans? Do they really have … ‘cause the CT scan is the worst. It, all these things worry me. All these scans. |
|  |  |  | Because it’s hit and miss on, on how long you can, kinda, live with it, you know what I mean? |
|  |  |  | ‘Cause I didn’t know, I don’t know how fast it’s growing or anything. I didn’t even know if I were gonna die in New York at one stage. I thought, I don’t feel poorly, there’s nothing amiss […] but you don’t, you just don’t know |
|  |  |  | I go for a scan on Friday […] this Friday, and the results are the day before my next treatment. So they’ll say whether it needs, they need to carry on with any treatment, or they don’t need to carry on […] So it’s a bit of a, that’s gonna be horrendous. |
|  |  |  | obviously, I don’t know what, I don’t know how, how bad mine is until the scan come, how bad or how good it is until the scan results come back, ‘cause the last, my last scan was in February. |
|  |  |  | When I’m at work I like, I like being, I like being at work ‘cause that’s also doing things normally and not having, being, having that mesothelioma on my mind. You know, just doing normal things with other people ‘cause that’s, when you’ve got something wrong with you, you don’t want to talk about it at all. You don’t want to talk about cancer, I hate hearing about cancer. |
|  |  |  | It’s the best way to not think about it now. |
|  |  | Brian | I can remember I was sitting in the chair, the professor was off to my right and the meso nurse was sitting on one of those bed things they have in, in the room. Was sitting on the edge of that. And when they started to explain to me what it was, and the enormity of it, it was that, that tunnel vision came in and everything closed down. And I've experienced it a few times in the past, and I know, and I know what it is. And it’s your whole world shrinking down to the, to what this is. |
|  |  |  | because of the, I think because of the, the having that constantly in the back of my mind, and because I have those extra, I had an extra two benefits coming in as a result of it. I, I basically gave up work. I, I, I really don't have the mental capacity or want to work thinking that I could spend the last year or last two years of my life working. |
|  |  |  | so I've got to constantly have that at the back of my mind. But I've got to have it as far at the back of my mind as I can have |
|  |  |  | There are times when we will have days when everything goes wrong. We catch every red light, the things that we want in the shops aren't there. Something shows up. And then I get something and go, oh yeah of course, not only is all that happening I’ve got this to deal with as well. It’s sort of like, it, it, it’s sort of like compounds other things, when things start to go wrong. |
|  |  |  | Even though, and it almost makes, makes you feel a bit like a leper, I suppose is the only word I can sort of like, it feels like you’re, you're sort of, you're there, even though you're fine in yourself, the people still need to know that you've, that you've got this for whatever reason. |
|  |  |  | But I was left in pretty much certain terms that it, it will come back. It will come back. It was explained that it is treatable, but it's not curable. So, if it does come back, they might be able to treat me again, they might not. |
|  |  |  | But I don't know how long I’ll have that for. I mean, do it, I could take out a great big loan and, and, and borrow loads of money to, to pay for it. But am I going to be around for long enough to pay the loan off? I don’t know. I plan for the future as if I'm going to have a future. […] But I don't know. And that's something that I've got to deal with. And unfortunately, so has my wife. |
|  |  |  | The solicitor actually send me a copy of the medical report from the doctor. The, the specialist doctor in court. And that was, that was quite interesting. But there was a one or two sombre bits in there and they did point out that I've got a more of a 50% chance that it will come back. And if it does, I'll probably be dead very painfully within two years. […] That was, it was pretty unpleasant reading that. |
|  |  |  | It's always in the back of my mind. You know, even and I’m, I'm more than happy to do this sort of thing. But even this sort of thing is just a little reminder that it is there. And so, there are, there are bits and pieces that, that are constant reminders. I mean, it's even things like I mean, that bless her, the, the, the lady that died of bowel cancer recently […] and they keep talking about cancer. And I can see why they do it. But then there's people like me sitting there going, yeah and that's what I’ve got and that could be me in a couple of years. And I have to sort of like, suppress that and put that to the back of my mind. |
|  |  |  | I was watching the news this morning, the first thing was about on, on BBC news, the first thing they were talking about was stillborn babies or where babies have died in the womb. And then they went on to talk about people that died from AIDS in the 80s. And then they went on about with Roy Castle, dying from lung cancer |
|  |  |  | it's a constant barrage of people dying of cancer and me sitting there thinking, yeah and, and that is going to be me at some stage. And it's those sort of things that I have to, if I, if I have the mental strength to push back to the back of my mind and, and acknowledge the fact that it’s there. I think for me, that's the biggest thing, whereas two years ago, it wouldn't have bothered me talking about all these deaths because that wasn't the, and it had nothing to do with my life. Whereas now to a certain degree it does. |
|  |  |  | Which when things that keep on about things with the best of intentions. Those are the little reminders, it's like we're not going to let you forget you've got this, we're not going to let you forget that you know there's a ticking time bomb in you. Because we're just going to keep reminding you of, of your mortality and, and, and everybody around you. And I think for me, if there was something that bothered me, it would be that. Is that, is that a reasonable? |
|  |  |  | It's just these constant reminders, that, that I think if, if it had to be anything, that's what it will be. |
|  |  |  | because of the fact that I'm, I've got these three-monthly scans, it’s actually has just been put back slightly to four monthly scans, I have to live my life in four months sections. So, it's a bit, it's a bit like the sword of Damocles, I'm just waiting for it to drop […] Because the chances are that whatever the, the next scan that I have, it could be its come back. If that's the case, the harsh reality is I could be dead within two years. |
|  |  |  | My son and daughter, they're like, they’re, the, we're living with it. We're all living with it. And, you know, every month or every. […] Four months now, sorry. Every four months now, they'll get a, a, a text message from, from the wife saying yeah, it’s all clear again, and then it’s yeah, okay. And on we go. So, as I say, we all live our life in four months sections. |
|  |  |  | so I've got to constantly have that at the back of my mind. But I've got to have it as far at the back of my mind as I can have |
|  |  |  | It's something that's there. It, it, it's a niggle that's there. And I think because I’ve, because it's been sort of like over a year now, I've, I’ve got to, I've had to get used to, to that niggle. |
|  | Subtheme 2.2 **Managing the Dread of Malnutrition** | Hannah | I think that a lot of peritoneal patients complain of digestive issues. So, I think it’s a common theme. |
|  |  |  | I did speak to one girl who she read something online to do with certain diets stopping cancer returning and things like that and she was trying to do this. I don’t even know what sort of diet it was but she, she was trying to … She said it was basically a lot of fruit and vegetables and little of anything else and she was trying to do that but after a month of that she gave up, because she just said it wasn’t sustainable you had to have a huge amount of time on your hands and it was really expensive. |
|  |  |  | And, and to be honest there’s no actual evidence that, that it does stop cancer from returning. So, so, yeah, you do hear things from people. But, yeah, there’s a lot of false information and I think that’s part of the problem. You do hear from people that have changed their diet because they don’t want cancer to return but in fact, like, they could actually be doing more harm than good, because there’s no evidence to support it. |
|  |  |  | A lot of the misinformation comes from US patients. I don’t know why but they seem to be more susceptible to believe, believing rubbish about, oh, how you cannot have cancer again if you quit sugar or, you know, whatever so, they, they, they seem a lot more, sort of, susceptible to believing that sort of thing and are more likely to listen to that sort of information online as well. |
|  |  |  | I mean, I was taking laxatives every day up to September. But then my, my gastro consultant turned round and said, “Oh, maybe you have a bacterial imbalance. Let’s try with antibiotics.” Had two weeks of antibiotics and not needed a laxative since. I wish someone had told me that, like, two years ago. because I had to put up with, like, six sachets of, of laxatives per day for, like, two years. So, it would have been nice to have known previously that that could have been fixed. |
|  |  |  | I ended up with chronic constipation. So, I had to start cooking foods that I wouldn’t normally have cooked before. Not saying that my diet was totally unhealthy, but it had to change to full of vegetables. Yeah, nothing sweet, because that seemed to provoke the liver problems. |
|  |  |  | it was, sort of, like, really learning how to, to go on from there. So, started cooking from recipe books and learnt a lot about nutrition and what things are useful and what things are not. I can’t eat rice, for example, rice just doesn’t agree with me for some reason I get a lot of indigestion, pains if I eat rice. I’ve, sort of, learnt along the way that, yeah, if my diet’s not particularly good I have a lot more stomach cramps. I get bloating, never really had these sort of problems before. So, yeah, I, I guess it’s along the same lines but it’s, it’s the sort of, the way that it changes everything. |
|  |  |  | I didn’t even know what chia seeds were before, before the operation and I’m going through, like, a packet a month. […] Like, you realise, like, raspberries are, like, really high fibre so, put them in a smoothie and they’re great. Don’t really tolerate milk very well so I switched to almond milk, been much better. Yeah, rice is a no and pasta, it’s okay but I tend to buy wholegrain pasta just because … or wholewheat pasta just so I feel that digestively it’s, it’s better but again, that has been a huge learning curve. |
|  |  | Evelyn | it’s a bit of a difficult one as well though because I’ve, I mean I love food and everything to do with food but part of the problems with the peritoneal is that it can cause, and in particular after the operation, lots of problems with eating. So it took a long time to get my, my, to, to sort of sort out problems afterwards because the, the operation kind of can cause a lot of digestive problems afterwards. |
|  |  |  | I think those that have had the operation can […] often have long term ongoing problems […] some people have to have part of the intestine removed and things and that can obviously cause further ongoing problems. |
|  |  |  | in terms of I should, I keep thinking I should improve my diet but at the same time I think well, actually keeping a bit of weight on might help me in the long term, you know, if I were to, if the disease was to reoccur. But I don’t know if that’s a healthy attitude really (laughs). |
|  |  |  | The food one is, is very much it’s sort of like how much, you know, I enjoy it and it’s, I mean there it was a social, you know, it’s a social experience. And it’s … But how, you know, how the disease has impacted me and might in the future with, with regard to food, so. |
|  |  |  | I suppose immediately after my operation, yes, I wouldn’t have been able to … And actually, just before I had my operation when I was feeling quite unwell and couldn’t, couldn’t eat much at all, you know, it made me realize how much, so much I, so much into social interaction was with, involved food really. So there was, there was a period just before my operation when I went out and other people were eating and I just couldn’t face eating. So, yeah, there was that sort of impact and yeah, it brought, a lot of it brought home to me how important it all is and how it has all tied in with it. |
|  |  |  | I think thinking about the future and, and how, and I mean, essentially think that it might be involved starving to death really if, if it does, the impact of peritoneal being that, that you just can’t get enough nutrition in that, that does frighten me a bit. You know, and I think that, that comes from, from, I don’t suppose people think about how actually how cancer kills you often and what, you know, what it is that actually kills you. And was highlighted again by my sister I think realizing that with her case it was liver disease, it was the… and the liver failure caused as a secondary cancer |
|  |  |  | So realizing that it’s not what you say, like with her case with it being bowel cancer it wasn’t that that killed her in the end. And I think it’s sort of like then thinking about how, yeah, how you die and how unpleasant it is… so the idea, yeah, of, of something that I really love, you know, food and eating is going to be something that I just won’t be able to do and then in the end |
|  |  | Sofia | food as well. I, like when you go through chemo some of the foods you get put off so, but then there are certain foods that I was just like totally addicted to. So I was like totally addicted to like curries and stuff like that and I still am. And I do a lot of food so we’re always making food, like nice things I always like treat myself. |
|  |  |  | Because one of things I got told like when I was going through therapy was that a one way of like how most people comfort themselves is like through the mouth so whether eating, drinking or like whatever. And I think that one of the ways that I soothe myself was by eating, not like overeating but like just making yourself feel |
|  |  |  | when you first get diagnosed people always have an opinion of, ‘Oh, say if you do this,’ or, ‘Say if you follow this diet,’ or ‘Say if you take this pill,’ or, ‘Say if you do this,’ and I just was like … And when you speak to the nurses, they’re like, ‘Go have that glass of wine, go eat that burger,’ because things like that are going to get you through and make me feel better than limiting yourself and eating, you could eat salad every night when you’re probably going through one of the worst things that you could go through |
|  |  |  | So this is a screenshot of a video I looked at like a plant based diet and I love curries so this is to represent, I do always believe that my plant based diet helped me a lot with my recovery after surgery. |
|  |  |  | So I kind of went back to my plant based, based diet and I do think it’s because it was easier to digest a lot of things like chickpeas and tofu and a lot of the stuff that I’m eating is not intolerable to my stomach so it was easier. Even when I was going through chemo, I still basically ate the same amount of food |
|  |  |  | So to me I think the plant based diet helped me a lot with my recovery and my eating. |
|  |  |  | I think it helped a lot with a lot of the, just because like our bodies aren’t meant to digest like meat and we’re not meant to, like dairy is quite intolerant to a lot of people. |
|  |  |  | Because like when you’re getting the surgery like they take all of your insides out and put it all back in again so they’re basically rebuilding a lot of stuff. So the food that I was eating was like stuff that was easy to digest in my stomach before it even gets to my bowel. Sorry, it’s going into graphic detail, but it was easier for me so and also, I was still getting all of the nutrients. |
|  |  |  | But for people maybe who are going through plant, non-plant based it might have been harder for them to digest. So I, I think it helped me a lot. |
|  |  |  | But yeah, it’s just, yeah, I do think that it helped me a lot. I lost a couple of kilos but I’ve, yeah, I’m fine now, so I don’t have any problem with it. |
|  |  |  | Yeah, it made me feel a lot better so like having that comfort, being able to eat something that I enjoy. But also, I think it helped with my strength as well so, because if you don’t eat and you don’t have the nutrients going in your body you’re not going to recover well. |
|  |  |  | So that would have made my physical, my mental health bad because I can’t eat. So and also, I think that helped me actually get through the chemo. So I never had any trouble getting through the chemo like my bloods were always fine and I always had the strength and I think that’s to do with the fact that I was eating fine |
|  |  | Faye | So you’ve got all this going round in your mind like is it going to grow? Am I going to need another operation? Oh my God is it going to stop my bowels working? Is it going to stop my stomach working? So that does swim around the whole time |
|  |  |  | but that’s tied up with, could I get travel insurance for it? […] Oh my God I don’t want to go anywhere if I can’t be covered for mesothelioma, you know, what happens if I get a bowel blockage and they say it’s caused by the operation. So, you know, there’s always something that you have to think about when you’ve got this. |
|  |  |  | the side effect of the surgery, my bowels aren’t brilliant, so I need to keep moving […] or else I just get bunged up. |
|  |  | Gladys | the only thing that, the one thing has changed is I don’t waste my time on eating food that I think is below par, you know. And I think that’s like a protective mechanism for myself. |
|  |  |  | But on a whole I do try and have as good food as I can and I don’t necessarily mean clean food, you know. But just good quality food with good ingredients. |
|  |  |  | because those are things that are completely within my control, aren’t they? What I put into my body and how I then use my body. |
|  |  |  | Because I’ve always been quite proactive in my health and wellbeing anyway, I’m not someone who likes a tablet. |
|  |  |  | recognize the importance of good quality sleep. […] And then we get back down to eating good food, keeping fit and healthy. |
|  |  |  | I do take … And I mean like I take vitamin D, I take, I’m a big firm believer of good healthy gut so I take quality pro and prebiotics, magnesium, I take fish oil stuff, omega stuff for my brain. |
|  |  | Clara | Because when his brother was ill, they kind of like wrote him off a little bit. And he reckons, like at the time, the one thing that kept him going was because he was still eating. So, I've been trying to like, you know, I don't want to like lose loads of weight, I want to try and build myself up before chemo and make sure I'm as strong as I can be. |
|  |  |  | I've been doing things to try and help. Like I've been, I have like these drinks that's got like, have got like ginger, turmeric, and all kinds of veggies and like anti-inflammatories and stuff like that. And yeah, it’s just certain foods. I was a vegan for like 15 years, but that's changed now. |
|  |  |  | So, I wanted to try and get a plate of food that's got the most like, nutritious value in it. So, I can even, like I can try and eat a bag of spinach, which isn't gonna happen, or I've gone to like fish and things, or I can try and eat things like that. So, just changed things to try and try and help myself a bit, really, and try and bring up you know, like a take on a lot of calories and things like that because I'm losing quite a lot of weight. |
|  |  |  | in like July and in August, I had a lump in my breast. So, I thought I had breast cancer. So, it turned out to be a cyst, luckily. So, I started doing the Gerson diet because my, my brain because I felt okay, like at that point, I felt fine. So, I think if you don't feel ill, you can then concentrate on like I've always done like exercise and things. And I just thought if I do this juice diet, this like Gerson and it's really like meant to be good for cancer and stuff, then that will just take my mind off it because they did the cancer, for two weeks the cancer pathway thing. So, for a couple of weeks, I just follow that religiously. And it took my mind off of everything |
|  |  |  | Yeah, just like his own way of like, things that he knows how to do. Just trying to make it appetising and because I've been following so many different like kind of diets, like on and off to try and like ease the IBS and the pain and stuff. Just trying to adapt things like food wise and make it appetising and still just keep bringing me things. |
|  |  | Natalie | I’ve just researched it massively, and, and just eating a lot more healthier |
|  |  |  | I researched having cancer a lot. Other than conventional treatments, obviously, what I’m doing, what else I could, before that started, what else I could do and look to research on, on, on what’s other people have done, done as well. And I found out that there is … obviously, I’ve not gone down that route, but there is a route you can go down where you totally change your, your diet and lifestyle, and it’s, and people have cured, cured their cancers or reduced tumours massively by literally just, it’s almost changing to a vegan diet. |
|  |  |  | And reducing all your, your toxins in your, you know, like, in your household. Like, I, so, so I’m doing some of the bits. So I filter tap water because there’s, you’ve got your fluoride and your chlorine in it, and stuff. It’s not good for you ‘cause I’m, I’m already putting my livers and kidney under a lot of pressure by having cancer and this chemotherapy without adding more toxins to my body. So, like, not going in traffic |
|  |  |  | And I just, I just read into it, and I’ve, and I’m on another group which a lot of people have cured, cured different types of cancers, not necessarily the cancer I’ve got, but they’ve reduced it or cured it just on, on not doing conventional, doing lifestyle and diet. So I have carrot, I have carrot juice every day. I found out that carrot juice, the juice from carrot is massively nutritious […] if you, instead of just eating a carrot, I mean, you eat them as well, but if you drink the juice, the, the, all the nutrition goes into your body. So I’m just doing that anyway. We’re, like, I juice two bags of carrots a day |
|  |  |  | So from about March time, I’ve been having carrot juices because there were these clinical trials that proved that when you pour it on cancer cells in the lab, it, it reduced it. And, like, garlic, all these super foods that you know, all those type of things, if you eat that in abundance it’s really good and it helps, it helps all your body become better at … because when you’ve got cancer you’re immune system is low. And what you’re trying to do is boost your immune system up massively to help fight, help, help, help kill the cancer cells. |
|  |  | Brian | I do try and make sure that the that food I eat is healthy foods. That's not to say that I eat healthily, and that I don't drink. But I try and make sure certainly, one of the things that I, I had a dietitian phone me up when I was really poorly before for the operation. […] And she told me to up my protein and I did up my protein. And I actually put a couple of pounds on doing that. So, I know that the protein, I gotta keep the protein up because it's the protein that’s gonna supply it. I mean, it’s, it's a bit like stating the obvious. |
|  |  |  | So, trying to make sure that I get enough protein in, in me as much as everything else. So, you know I might eat loads of chocolate and drink loads of beer, which is loads of calories. But I also got to make sure I get loads of protein in amongst those calories. |
|  |  |  | it's not a case of calorie counting, it's just a case of no matter what I eat, I've got much to all of this chicken in there, or there's tuna in there, or there's, you know that that, that I mean, breakfast was a protein shake. I shoved a load of peanut butter in as well. And so, I know that there's, there’s plenty of protein in there. So regardless of what else I eat and drink during the day. I mean, I'd have chicken again this evening. What I eat or drink, no matter how bad it might be for me, at least I know the proteins in there with it. |
